# In Vitro Antifungal Activity and Toxicity of Dihydrocarvone-Hybrid Derivatives against *Monilinia fructicola*

**DOI:** 10.3390/antibiotics10070818

**Published:** 2021-07-06

**Authors:** Katy Díaz, Enrique Werner, Ximena Besoain, Susana Flores, Viviana Donoso, Bastian Said, Nelson Caro, Ernesto Vega, Iván Montenegro, Alejandro Madrid

**Affiliations:** 1 Departamento de Química, Universidad Técnica Federico Santa María, Av. España N° 1680, Valparaíso 2340000, Chile; katy.diaz@usm.cl; 2 Departamento de Ciencias Básicas, Campus Fernando May, Universidad del Bío-Bío, Avda. Andrés Bello 720, Casilla 447, Chillán 3780000, Chile; ewerner@ubiobio.cl; 3 Escuela de Agronomía, Pontificia Universidad Católica de Valparaíso, San Francisco s/n La Palma, Quillota 2260000, Chile; ximena.besoain@pucv.cl; 4 Laboratorio de Productos Naturales y Síntesis Orgánica (LPNSO), Departamento de Química, Facultad de Ciencias Naturales y Exactas, Universidad de Playa Ancha, Avda. Leopoldo Carvallo 270, Playa Ancha, Valparaíso 2340000, Chile; susana.flores@upla.cl (S.F.); cadonoso@utalca.cl (V.D.); 5 Departamento de Química, Universidad Técnica Federico Santa María, Av. Santa María 6400, Santiago 7630000, Chile; bastian.said@usm.cl; 6 Centro de Investigación Australbiotech, Universidad Santo Tomás, Avda. Ejército 146, Santiago 8320000, Chile; ncaro@australbiotech.cl; 7 Departamento Laboratorios y Estaciones Cuarentenarias, Servicio Agrícola y Ganadero, Ruta 68 # (Km. 12), Pudahuel 9020000, Chile; ernesto.vega@sag.gob.cl; 8 Escuela de Obstetricia y Puericultura, Facultad de Medicina, Universidad de Valparaíso, Angamos 655, Reñaca 2520000, Chile; ivan.montenegro@uv.cl

**Keywords:** dihydrocarvone, *Monilinia fructicola*, hybrid compounds

## Abstract

The aim of this study was to synthesize a series of novel and known dihydrocarvone-hybrid derivatives (**2**–**9**) and to evaluate mycelial growth activity of hybrid molecules against two strains of *Monilinia fructicola*, as well as their toxicity. Dihydrocarvone-hybrid derivatives have been synthesized under sonication conditions and characterized by FTIR, NMR, and HRMS. Antifungal efficacy against both strains of *M. fructicola* was determined by half maximal effective concentration (EC_50_) and toxicity using the brine shrimp lethality test (BSLT). Among the synthesized compounds, **7** and **8** showed the best activity against both strains of *M. fructicola* with EC_50_ values of 148.1 and 145.9 µg/mL for strain 1 and 18.1 and 15.7 µg/mL for strain 2, respectively, compared to BC 1000^®^ (commercial organic fungicide) but lower than Mystic^®^ 520 SC. However, these compounds showed low toxicity values, 910 and 890 µg/mL, respectively, compared to Mystic^®^ 520 SC, which was highly toxic. Based on the results, these hybrid compounds could be considered for the development of more active, less toxic, and environmentally friendly antifungal agents against phytopathogenic fungi.

## 1. Introduction

The fresh fruit industry is the fastest growing agricultural sector worldwide in terms of production, exports, and employment generation [1]. In this context, stone fruits (e.g., peaches, nectarines, plums, apricots, and cherries) are an important fruit crops and play a significant role in the food and nutritional security [2]. Unfortunately, there are various production impediments in stone fruit cultivation, of which diseases are considered as the major limiting factors, which affect the yield and quality of the fruits, thus making their cultivation less remunerative [3]. Brown rot disease, which is caused by the pathogenic fungi of *Monilinia fructicola* (G. Winter) Honey (phylum Ascomycota, family Sclerotiniaceae), is a leading constraint in world stone fruit production [4]. The high incidence of plant mortality and the lack of effective control methods cause billions of dollars in losses worldwide each year [5]. Hence, great efforts and various management strategies have been made to control brown rot and minimize the severity of this pathology [6].

Brown rot is managed mainly by the periodic application of synthetic fungicides, from flowering to the pre- and postharvest stages [7]. However, resistance to benzimidazoles, dicarboximide, and IBS fungicides is frequently documented [8,9,10]. Despite all of the benefits that fungicides bring in controlling brown rot, their use is controversial and they are not allowed during postharvest stages because they can be harmful to consumer health [11].

Several chemical structures have been found effective in killing or inhibiting the microbial growth; these include terpene [12] and chalcone derivatives [13]. Some terpene derivatives have been found with a broad spectrum of antimicrobial activity [14]. Among this group of molecules, terpenic ketones, such as dihydrocarvone, are important subclass of natural compounds, which can serve as versatile synthetic building blocks and key intermediates in drug design [15]. Dihydrocarvone is the main component in the essential oil from *Poiretia latifolia* flowers (50–70% present) [16] and is present in low proportions in other oils with antifungal capacity, such as *Mentha longifolia*, *M. spicata*, *M. pulegium*, and *Anethum sowa* [17,18,19]. This monoterpene ketone is a potential growth inhibitor of yeast fungi such as *Saccharomyces cerevisiae*, *Candida albicans*, and *Cryptococcus neoformans* [16]. On the other hand, chalcone derivatives are also well known antimicrobial agents [20]. In addition, many chalcone scaffold antifungal agents have been well investigated [21].

Multifunctional features of these two groups of natural compounds make them ideal blocks for hybrid scaffolds with interesting antifungal properties. In this context, exocyclic unsaturated ketones are an important pharmacophore from natural plants, and many natural molecules (such as flavonoids, quinolines, cinnamic acid derivatives, etc.) have been discovered with this unit; these are suitable starting materials for the synthesis of versatile heterocycles with polycyclic skeletons [22]. Some of the exocyclic unsaturated ketones, namely, benzylidene-cycloalkanones, are an important class of hybrid compounds that have received significant attention for the past few decades because of the broad spectrum of their applications in biochemical, pharmaceutical, material, and agrochemical sciences [22,23].

We report herein the synthesis of dihydrocarvone-hybrid derivatives and study their antifungal and toxicity activities against two strains of *M. fructicola* in order to explain the possibility of such compounds to act either as control agents of postharvest fungal pathogens.

The aim of this study is to synthesize eight benzylidene-cycloalkanones derivatives and to study their antifungal activity against *M. fructicola* in vitro assays. Skeleton structures of new derivatives were synthesized via Claisen–Schmidt condensation. The compounds were assayed of inhibition of mycelial growth.

## 2. Results and Discussion

### 2.1. Chemistry

**Scheme 1:**
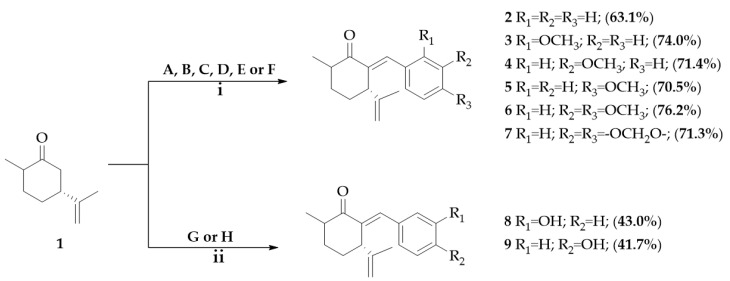
General scheme for the synthesis of dihydrocarvone-hybrid derivatives **2**–**9**. Reagents and conditions: (**i**) KOH (20%), MeOH, US irradiation at 25–35 °C for 3 h; (**ii**) Yb(OTf)_3_ (0.1 mol), US irradiation at 55–65 °C for 3–5 h.

**Figure 1:**
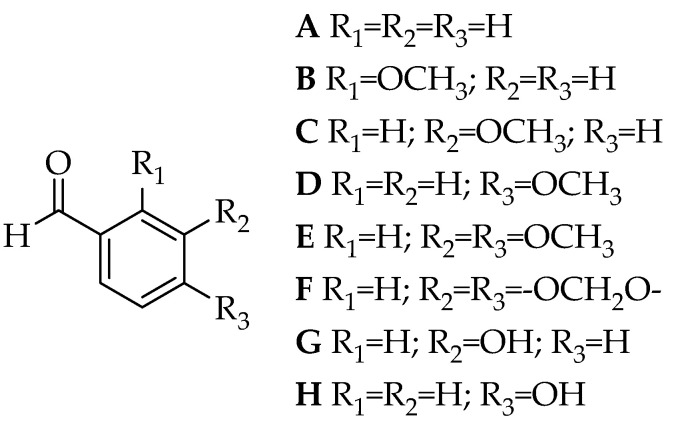
Structure of aromatic benzaldehydes.

Dihydrocarvone (**1** on Scheme 1) and different aromatic aldehydes **A**–**H** (Figure 1) were used as starting materials for the synthesis of dihydrocarvone-hybrid derivatives.

The method for synthesis the target compounds is shown in Scheme 1 in a condition as previously reported [24] with modifications. Dihydrocarvone-hybrid derivatives **2**–**9** were obtained in moderate to good yields (45.2–76.2%).

Our initial efforts were focused on carrying out the synthesis of this type of monoarylidene cycloalkanones for the first time and optimizing the synthesis parameters such as catalyst, reaction time, and yield. Mainly due to the poor reactivity of monoterpene cyclic ketones, such as dihydrocarvone, under normal reaction conditions, such as, e.g., the use of strong bases, polar aprotic solvents, at reflux and for long reaction times, we used DMSO as solvent and *t*-BuOK as base [24], since the described combination was the best for this type of molecules and there was no reaction.

For this purpose, the series of hybrid compounds were synthesized via Claisen–Schmidt condensation using KOH as a catalyst under ultrasound irradiation [25] for benzaldehyde and alkyl-substituted aromatic aldehydes. In the case of benzaldehydes with free hydroxyl groups (**G**–**H**), which show low reaction yields (**8** (1.7%) and **9** (2.5%)) because couplings with free hydroxyl groups are difficult for many reasons, including their relatively low nucleophilicity [26] compared to other functional groups such as ethers and halogens, these were subjected to a Yb(OTf)_3_ catalyzed condensation under solvent-free conditions [27] under ultrasound irradiation, obtaining with this method the desired compounds (**8** and **9**) with better yields (51.0% and 53.7%, respectively) than yields from traditional methods [24]. Furthermore, the use of ultrasound irradiation can lead to improved reaction efficiency, i.e., increased yields and reduced reaction time [28]. Additionally, in many cases, reactions under ultrasound irradiation represent more environmentally friendly processes, free of solvents or with only small amounts of solvents, all while consuming less energy [29].

**Figure 2:**
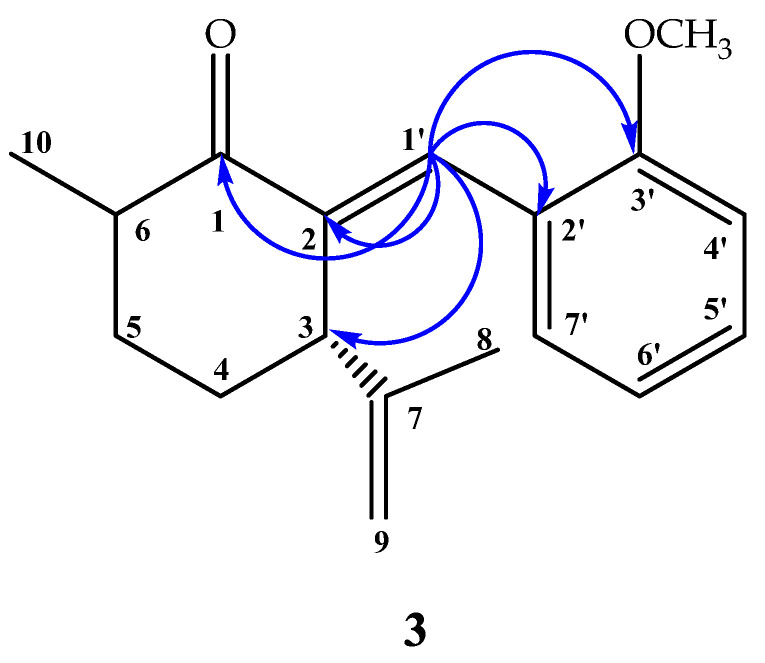
Representative HMBC correlations of compound **3**.

On the basis of NMR, the structures of all synthesized molecules were determined (Figure S1). Singlet signals with chemical shifts in the range of 7.67–7.41 ppm (s, 1H) and 137.3–130.0 ppm for ^1^H and ^13^C spectra, respectively, were observed in the ^1^H spectrum of synthetic compounds **2**–**9** and were attributed to the presence of typical trans-olefinic proton, corresponding to the bond between the cycloalkanone and the aromatic ring, values that are in agreement with those previously reported for this family of compounds [24,30,31]. These data were corroborated for all the molecules using the heteronuclear multiple-bond correlation (HMBC) spectra. In general, the H-1′ of the dihydrocarvone-hybrid derivatives showed heteronuclear couplings at *^2^ J* and *^3^ J* with the carbon 2 and 2′ and the carbons 1, 3, and 3′, respectively. An example of these interactions can be seen in compound **3** (Figure 2).

### 2.2. Biological Activities

#### 2.2.1. Antifungal Activity of Dihydrocarvone-Hybrid Derivatives against Both Strains of *Monilinia fructicola* In Vitro

The antifungal activity of the compounds dihydrocarvone-hybrid derivatives against strains 1 and 2 of the pathogenic fungus *M. fructicola* evaluated through the radial growth test indicates that there is a directly proportional effect, that is, as we increase the concentration of the compounds, the inhibition of mycelial growth increases.

**Figure 3:**
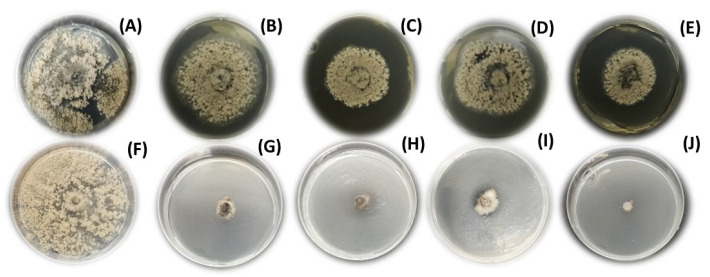
Effect of compound **8** on in vitro mycelial growth of strains 1 (**A**–**E**) and 2 (**F**–**J**) of *M. fructicola.* Negative control (0 µg/mL) (**A**,**F**); 150 µg/mL (**B**,**G**); 250 µg/mL (**C**,**H**); 250 µg/mL positive control (organic commercial fungicide BC-1000^®^) (**D**,**I**); 250 µg/mL positive control (commercial fungicide Mystic^®^ 520 SC) (**E**,**J**).

The results depicted in Figure 3, show that compound **8** has superior antifungal activity against both strains of *M. fructicola*, presenting inhibition percentages within a range of 47–88% at 150 µg/mL and 62–97% at 250 µg/mL, which shows that both strains respond to the fungicidal action of the compound; however, strain 2 is more sensitive than strain 1 for all compounds. Like the other two active compounds **3** and **7**, these molecules are similar in their variation and incorporation of methoxyl and dioxymethylene groups in the C3 position of the aromatic ring, respectively, which is in agreement with other authors, indicating that this modification is decisive when designing structures with antimicrobial biological effect [32].

If we compare the antifungal activity between both positive controls, it is observed that the commercial synthetic fungicide is more active than the commercial organic fungicide BC-1000^®^; however, as these compounds are derived from the hybridization of two organic molecules (monoterpene ketone plus a series of benzaldehydes, all of natural origin), the positive control BC 1000^®^ will be used to statistically compare the effective concentration doses (EC_50_) (Table 1).

The EC_50_ values for all assayed compounds are given in Table 1. The screening of the antifungal activities of dihydrocarvone-hybrid derivatives (**2**–**9**) on the strains 1 of *M. fructicola* revealed that the molecules showed no significant ability to inhibit the growth of the fungus. However, two (**7** and **8**) of the three compounds that inhibit the mycelial growth of the strains 1 of *M. fructicola* are significant when compared with the commercial organic fungicide, reducing its effective dose concentration to half in a range of 145.9–148.1 µg/mL. It is interesting to note that this is the first report of antifungal activity against this phytopathogen of this type of compounds.

In contrast, the antifungal activity against strain 2 presents compounds with lower EC_50_ values than strain 1, close to the response of the organic positive control, improving the growth inhibition action with significantly lower EC_50_ values of 15.7–23.1 µg/mL of compounds **5**, **7**, and **8** to achieve control of mycelial growth of the pathogen.

It is expected that the discovery of these molecules will be a precedent for the development of new molecules modifications mainly on the exocyclic double bond of the natural ketone, which would further enhance its antifungal effect [33,34,35]. Our findings show that compounds **7** and **8** are capable of combating highly resistant strains such as strain 1 at higher concentrations, which would allow combating more aggressive strains that cause severe crop damage at flowering time. This difference in susceptibility to the same dihydrocarvone-hybrid derivatives molecules may be due to modifications of the target site of the strains, as this is the most common cause of fungicide resistance [36].

The emergence and spread of dual-resistant strains of *M. fructicola* could have implications for this disease management strategy [37] consequences for this disease management strategy, so it is necessary to focus our efforts on generating hemisynthetic fungicides with a broad spectrum of action.

The compounds **7** and **8** are capable of combating fewer sensitive strains such as strain 1, which would allow combating more aggressive strains that cause severe crop damage at flowering time.

#### 2.2.2. Cytotoxic Activity

The cytotoxic activity of the compounds **1**–**9** was evaluated using a brine shrimp lethality test (BSLT) method as a preliminary test (Table 2).

BSLT is an efficient, rapid, and inexpensive test that requires only a relatively small amount of sample. This bioassay has a good correlation with pesticide activity and has led to the discovery of a number of new molecules categorized as natural pesticides [38,39,40]. The results obtained in BSLT, indicate that compounds **2**–**5** are considered weak or nontoxic (compounds > 1000 µg/mL) and compounds **6**–**9** weak to moderate toxicity (values between 100 and 1000 µg/mL) [41,42], but compound **9** is more toxic in comparison with the others compounds because it needs a lower concentration to achieve 50% mortality. However, it is less toxic than the natural starting compound, 80 times less than the commercial control of *M. fructicola*. Therefore, the active synthesized compounds could be used as potential postharvest antifungal agents. In addition, biological effects (especially phytotoxicity) of derivative compounds will be studied in stone fruits plants. The compounds with the best antifungal activity will be loaded into a series of nanoemulsions to improve their bioavailability and lower the effective dose against *M. fructicola*. This study will also vary experimental laboratory conditions to examine the effect of nanoemulsion growth inhibition against *M. fructicola* and, in greenhouse and field conditions, the preventive or curative effects on plant and tissues in vivo.

## 3. Materials and Methods

All solvents, chemicals, and reagents were obtained commercially from Sigma-Aldrich Co. (St. Louis, MO, USA) and used without purification. The compounds **2**–**9** were isolated and characterized as previously reported [43].

### 3.1. Synthesis

#### 3.1.1. Synthesis of Dihydrocarvone-Hybrid Derivatives 2–7

A mixture of dihydrocarvone (**1**) (3.28 mmol) and commercial benzaldehyde **A**–**F** (1.2 molar equivalents) was taken in a 100 mL round-bottom flask. Both reagents were solubilized in methanol (5 mL), a KOH solution was added (in 5 mL of methanol), and the mixture was irradiated in the water bath of an ultrasonic cleaner at 25–35 °C for 3 h. Sonication was performed in water bath of an ultrasonic cleaner (Elmasonic S 10 H, Elma Schmidbauer GmbH, Sigen, Germany) with a frequency of 25 kHz and a nominal power 400 W. The reaction flask was located in the maximum energy area in the cleaner, and the addition or removal of water controlled the temperature of the water bath. Then, the mixture was cooled in an ice-water bath, after which 5% HCl solution was added until pH ~ 7 to end the reaction, and the mixture was extracted with EtOAc (3 × 30 mL). The organic layer was dried with Na_2_CO_3_, filtered, and separated with column chromatography using a hexane/EtOAc mixture increased polarity, obtaining compounds 2–7 in yields between 63.1% and 76.2%.

(3S)-2-benzylidene-3-isopropenyl-6-methylcyclohexanone (**2**): Pale yellow oil. Spectroscopic data of compound **2** were consistent with those reported in the literature [44].

(3S)-3-isopropenyl-2-(2-methoxybenzylidene)-6-methylcyclohexanone (**3**): Yellow oil. [α]_D_^22^ = −1.76 (c = 0.17 g/mL, CH_2_Cl_2_); FTIR (υmax cm^−1^): 2949, 2360, 1730; 1450; 1290. ^1^H NMR (400 MHz, CDCl_3_): δ 7.44 (s, 1H, H-1′); 7.28 (m, 1H, H-7′); 7.21 (d, *J* = 7.2 Hz, 1H, H-4′); 6.88 (m, 2H, H-5′ and H-6′); 5.00 (s, 1H, H-9_β_); 4.67 (s, 1H, H-9_α_); 3.82 (s, 3H, OCH_3_); 3.55 (s, 1H, H-3); 2.36 (m, 1H, H-6); 2.10–1.79 (m, 2H, H-4); 1.87–1.62 (m, 2H, H-5); 1.82 (s, 3H, H-8); 1.17 (d, *J* = 3.5 Hz, 3H, H-10). ^13^C NMR (100 MHz, CDCl_3_): δ 205.88 (C-1); 158.1 (C-3′); 146.4 (C-7); 130.0 (C-1′); 129.9 (C-5′); 129.4 (C-7′); 124.7 (C-2′); 120.0 (C-6′); 114.9 (C-8); 110.3 (C-4′); 55.4 (OCH_3_); 46.0 (C-3); 45.4 (C-6); 28.3 (C-5); 27.0 (C-4); 21.5 (C-9); 15.9 (C-10). HRMS-ESI: [M + 1]^+^ 271.1676 (m/z calcd for C_18_H_22_O_2_, 270.1620).

(3S)-3-isopropenyl-2-(3-methoxybenzylidene)-6-methylcyclohexanone (**4**): Yellow oil. [α]_D_^22^ = −0.92 (c = 0.27 g/mL, CH_2_Cl_2_); FTIR (υmax cm^−1^): 2954, 2357, 1712, 1454, 1310. ^1^H NMR (400 MHz, CDCl_3_): δ 7.60 (s, 1H, H-1′); 7.27 (m, 1H, H-6′); 6.96 (m, 1H, H-3′); 6.93 (m, 1H, H-7′); 6.89 (m, 1H, H-5′); 5.01 (s, 1H, H-9_β_); 4.71 (s, 1H, H-9_α_); 3.79 (s, 3H, OCH_3_); 3.66 (s, 1H, H-3); 2.38–1.82 (m, 3H, H-4 and H-6); 1.86 (s, 3H, H-8); 1.46–1.28 (m, 2H, H-5); 1.25 (s, 3H, H-10). ^13^C NMR (100 MHz, CDCl_3_): δ 206.4 (C-1); 159.9 (C-4′); 144.9 (C-2); 140.2 (C-7); 137.3 (C-1′); 136.3 (C-2′); 129.5 (C-6′); 122.4 (C-7′); 115.6 (C-8); 114.3 (C-5′); 109.7 (C-3′); 55.2 (OCH_3_); 45.8 (C-3); 44.9 (C-6); 29.7 (C-5); 29.7 (C-4); 21.5 (C-9); 15.9 (C-10). HRMS-ESI: [M + 1]^+^ 271.1685 (m/z calcd for C_18_H_22_O_2_, 270.1620).

(3S)-3-isopropenyl-2-(4-methoxybenzylidene)-6-methylcyclohexanone (**5**): Yellow oil. [α]_D_^22^ = −0.86 (c = 0.29 g/mL, CH_2_Cl_2_); FTIR (υmax cm^−1^): 2918, 2360, 1734, 1500, 1297. ^1^H NMR (400 MHz, CDCl_3_): ^1^H NMR (400 MHz, CDCl_3_): δ 7.60 (s, 1H, H-1′); 7.26 (m, 2H, H-3′ and H-7′); 6.90 (m, 2H, H-4′ and H-6′); 5.01 (s, 1H, H-9_β_); 4.71 (s, 1H, H-9_α_); 3.78 (s, 3H, OCH_3_); 3.65 (s, 1H, H-3); 2.03 (m, 1H, H-6); 1.91–1.81 (m, 2H, H-4); 1.87 (s, 3H, H-8); 1.45-1.28 (m, 2H, H-5); 1.25 (s, 3H, H-10). ^13^C NMR (100 MHz, CDCl_3_): δ 203.2 (C-1); 159.4 (C-5′); 145.8 (C-2); 139.1 (C-7); 136.5 (C-1′); 129.3 (C-3′ and C-7′); 122.6 (C-2′); 115.1 (C-8); 113.9 (C-4′ and C-6′); 55.3 (OCH_3_); 44.9 (C-3); 31.9 (C-6); 29.7 (C-5); 29.3 (C-4); 21.5 (C-9); 14.1 (C-10). HRMS-ESI: [M + 1]^+^ 271.1679 (m/z calcd for C_18_H_22_O_2_, 270.1620).

(3S)-2-(3,4-dimethoxybenzylidene)-3-isopropenyl-6-methylcyclohexanone (**6**): Yellow oil. [α]_D_^22^ = −1.15 (c = 0.13 g/mL, CH_2_Cl_2_); FTIR (υmax cm^−1^): 2943, 2360, 1701, 1502, 1305. ^1^H NMR (400 MHz, CDCl_3_): ^1^H NMR (400 MHz, CDCl_3_): δ 7.67 (s, 1H, H-1′); 7.26 (m, 1H, H-3′); 7.00 (m, 1H, H-7′); 6.87 (m, 1H, H-6′); 5.02 (s, 1H, H-9_β_); 4.75 (s, 1H, H-9_α_); 3.90 (s, 3H, OCH_3_); 3.85 (s, 3H, OCH_3_); 3.66 (s, 1H, H-3); 2.01 (m, 1H, H-6); 1.92–1.28 (m, 4H, H-4 and H-5); 1.89 (s, 3H, H-8); 1.25 (m, 3H, H-10). ^13^C NMR (100 MHz, CDCl_3_): δ 203.3 (C-1); 150.1 (C-4′); 148.6 (C-5′); 145.5 (C-2); 140.1 (C-7); 134.7 (C-1′); 128.0 (C-2′); 123.4 (C-7′); 114.9 (C-8); 113.0 (C-6′); 110.9 (C-3′); 55.9 (OCH_3_); 55.7 (OCH_3_); 44.9 (C-3); 32.5 (C-6); 29.7 (C-5); 29.3 (C-4); 21.5 (C-9); 14.1 (C-10). HRMS-ESI: [M + 1]^+^ 301.1806 (m/z calcd for C_19_H_24_O_3_, 300.1725).

(3S)-2-(1,3-benzodioxol-5-ylmethylene)-3-isopropenyl-6-methylcyclohexanone (**7**): Dark yellow oil. [α]_D_^22^ = -3.72 (c = 0.47 g/mL, CH_2_Cl_2_); FTIR (υmax cm^−1^): 2893, 2359, 1684, 1489. ^1^H NMR (400 MHz, CDCl_3_): δ 7.58 (s, 1H, H-1′); 6.91 (d, *J* = 8 Hz, 1H, H-7′); 6.85 (m, 1H, H-6′); 6.79 (s, 1H, H-3′); 5.99 (s, 3H, OCH_2_O); 4.98 (s, 1H, H-9_β_); 4.70 (s, 1H, H-9_α_); 3.66 (s, 1H, H-3); 2.03 (m, 1H, H-6); 1.91–1.28 (m, 4H, H-4 and H-5); 1.89 (s, 3H, H-8); 1.25 (m, 3H, H-10). ^13^C NMR (100 MHz, CDCl_3_): δ 203.3 (C-1); 148.4 (C-5′); 147.7 (C-4′); 145.4 (C-2); 139.5 (C-7); 136.6 (C-1′); 129.3 (C-2′); 125.0 (C-7′); 115.4 (C-8); 109.7 (C-6′); 108.4 (C-3′); 101.4 (OCH_2_O); 44.7 (C-3); 32.5 (C-6); 29.7 (C-5); 29.3 (C-4); 21.4 (C-9); 14.1 (C-10). HRMS-ESI: [M + 1]^+^ 285.1480 (m/z calcd for C_18_H_20_O_3_, 284.1412).

#### 3.1.2. Synthesis of Dihydrocarvone-Hybrid Derivatives 8 and 9

A mixture of dihydrocarvone (**1**) (3.28 mmol) and commercial benzaldehyde **G** and **H** (1.2 molar equivalents) and Yb(OTf)_3_ (0.025 mmol, 0.5 mol%) was taken in 100 mL round-bottom flask. The mixture was irradiated in the water bath of an ultrasonic cleaner at 55–65 °C for 5 h. After the reaction was complete, the system was cooled to r.t.; then, the reaction was diluted with 10 mL alcohol and 15 mL deionized water, and the contents were stirred for 10 min. Later, the mixture was extracted with EtOAc (3 × 30 mL). The catalyst remaining in the aqueous phase was recovered by removing the water by heating and then drying under reduced pressure at 100 °C for 2 h and the organic layer was dried with Na_2_CO_3_, filtered, and separated with column chromatography using a hexane/EtOAc mixture increased polarity, obtaining compounds **8** and **9** in yields between 45.2% and 53.7%.

(3S)-2-(3-hydroxybenzylidene)-3-isopropenyl-6-methylcyclohexanone (**8**): Orange viscous oil. [α]_D_^22^ = −1.42 (c = 0.70 g/mL, CH_2_Cl_2_); FTIR (υmax cm^−1^): 3310, 2924, 2362, 1695, 1593. ^1^H NMR (400 MHz, CDCl_3_): δ 7.41 (s, 1H, H-1′); 7.26 (s, 1H, H-3′); 7.21 (m, 1H, H-6′); 6.86 (m, 1H, H-7′); 6.81 (m, 1H, H-5′); 4.99 (s, 1H, H-9_β_); 4.65 (s, 1H, H-9_α_); 3.66 (s, 1H, H-3); 2.35 (m, 1H, H-6); 2.02 (m, 1H, H-4_b_); 1.86–1.77 (m, 1H, H-4_a_); 1.64 (m, 1H, H-5_b_); 1.33-1.25 (m, 1H, H-5_a_); 1.17 (d, *J* = 6.6 Hz; 3H, H-10). ^13^C NMR (100 MHz, CDCl_3_): δ 206.4 (C-1); 155.7 (C-4′); 145.9 (C-2); 141.6 (C-7); 137.0 (C-1′); 134.9 (C-2′); 129.5 (C-6′); 121.7 (C-7′); 116.4 (C-5′); 115.7 (C-8); 115.1 (C-3′); 45.8 (C-3); 45.5 (C-6); 28.1 (C-5); 27.0 (C-4); 21.5 (C-9); 16.0 (C-10). HRMS: [M + 1]^+^ 257.1534 (m/z calcd for C_17_H_20_O_2_, 256.1463).

(3S)-2-(4-hydroxybenzylidene)-3-isopropenyl-6-methylcyclohexanone (**9**): Brown viscous oil. Spectroscopic data of compound **9** were consistent with those reported in the literature [45].

### 3.2. Biological Activities

#### 3.2.1. Antifungal Activities of Dihydrocarvone-Hybrid Derivatives against *M. fructicola* In Vitro

Both strains (S1 and S2) of *M. fructicola* were kindly provided by the phytopathology laboratory of the Servicio Agrícola y Ganadero de Chile (SAG), Santiago, Chile. The isolate S1 was recovered from infected peach from commercial orchards in the O’Higgins region, Chile, and the isolate S2 was recovered from infected nectarines from commercial orchards in the province of Maipo, Metropolitan Region, Chile and identified to species level with a PCR assay developed by the molecular biology laboratory belonging to SAG; these were maintained on potato dextrose agar (PDA) and incubated for 120 h at 24 °C.

The antifungal activity of all dihidrocarvone-hybrid derivatives (**2**–**9**) against both strains of *M. fructicola* was determined by the radial test previously reported [46]. The test compounds were dissolved in ethanol (5% *v*/*v*) and added to the potato dextrose agar medium (PDA) medium in the petri dishes to obtain a final concentration of 10, 25, 50, 150, and 250 µg/mL. The mycelial growth diameters were measured after 120 h of incubation at 24 °C in the dark, and the inhibition percentages were calculated with respect to the negative ethanol control. The commercial fungicides Mystic^®^ 520 SC (Pyrimethanil 400 g/L, Trifloxystrobin 120 g/L) (Lot: PAIS004727; Bayer, Santiago, Chile) and BC-1000^®^ (grapefruit seed and pulp extract (*Citrus x paridisi*), 50% *w*/*v* (500 g/L)) were used as positive controls and were measured under the same conditions as the compounds.

The results were expressed as the effective concentration (EC_50_) that reduced mycelial growth by 50%. This value was determined by regressing the values of the percentage inhibition of radial growth against the compound concentration values. The fit analysis was performed using the Origin Pro^®^ V. 8.0 software (OriginLab Corporation, Northampton, Massachusetts, EE.UU) [46]. These experiments were performed in triplicate and each assay was performed twice (*n* = 6). Significant differences were determined using a one-way analysis of variance, followed by a pairwise comparison of means (LSD test; *p* < 0.05) (Microsoft Office Excel^®^ 2016).

#### 3.2.2. Brine Shrimp Lethality Test

The assay was carried out according to the principle and protocol previously described [47,48]. Briefly, *Artemia salina* L. eggs were inserted into a box containing seawater; the box was placed under a UV lamp; after 48 h, the eggs hatched into larvae and were ready for the test. All of the compounds were dissolved in methanol at final concentrations of 1000 to 10 µg/mL. After 24 h, the live and dead shrimp were counted. The experiment was conducted in triplicate. The median lethal concentrations (LD_50_) with 95% confidence intervals were determined using the Probit analysis method. For LC_50_ values, probit analysis was used with Minitab V. 15 software (Minitab^®^ Statistical Software, State College, PA, USA).

## 4. Conclusions

The results suggest that studied dihydrocarvone-hybrid derivatives have potential as new antifungal agents against *M. fructicola*. It can also be concluded that the choice of acidic or basic medium is key in condensation due to the nature of the aldehyde substituent, which plays a key role since the ultrasound-assisted one-step protocol led to better yield of benzylidene-cycloalkanones. There is a difference in sensitivity to these compounds, with strain 2 being more sensitive than strain 1. Furthermore, compounds **7** and **8** showed the major antifungal effect of hybrid derivatives for both strains. These new compounds could be successful in the control of other fungal phytopathogens closely related to members of the Sclerotiniaceae family such as *Botrytis cinerea*, *B. aclada*, *Sclerotinia sclerotiorum*, *S. cepivorum*, and *Ciborinia camelliae*. Future studies could validate dihydrocarvone-hybrid derivatives as useful new fungicides for fruit production industry.

## Figures and Tables

**Table 1:** EC_50_ values of dihydrocarvone-hybrid derivatives (**2**–**9**) on the in vitro mycelial growth of both strains of *Monilinia fructicola*.

	Strains			
	S1		S2	
**Compound**	EC_50_ ± SD (µg/mL)	*R*	EC_50_ ± SD (µg/mL)	*R*
**1**	i	i	i	i
**2**	i	i	i	i
**3**	i	i	58.0 ± 0.7	0.9101
**4**	273.2 ± 0.9	0.8859	39.6 ± 1.1	0.9341
**5**	i	i	23.1 ± 1.2	0.9592
**6**	i	i	47.7 ± 0.8	0.8831
**7**	148.1 ± 1.4	0.9856	18.1 ± 0.0	0.9781
**8**	145.9 ± 1.7	0.9580	15.7 ± 1.3	0.9703
**9**	i	i	i	i
**Mystic^®^ 520 SC**	33.73 ± 1.0	0.8914	9.1 ± 0.0	0.9998
**BC-1000^®^**	280.0 ± 1.6	0.9500	10.5 ± 0.1	0.9884
**Negative Control**	i	i	i	i

EC_50_: concentration causing 50% mycelial growth inhibition; SD: standard deviation; *R:* Pearson’s. Letters in superscript indicate significant differences (*p* > 0.05) compared with BC-1000^®^ (*n* = 6).

**Table 2:** BSLT results for dihydrocarvone-hybrid derivatives **1**–**9**.

Compound	LD_50_ (µg/mL)	95% CI ^a^
**1**	550	384–918
**2**	>1000	nd
**3**	>1000	nd
**4**	>1000	nd
**5**	>1000	nd
**6**	954	580–1878
**7**	910	822–1537
**8**	890	533–1757
**9**	847	478–2244
**Mystic^®^ 520 SC**	10.3	9.9–15.6

^a^ 95% confidence interval, nd: (not determined).

## Data Availability

All data are available for the scientific community.

## Supplementary Materials

The following are available online at https://www.mdpi.com/article/10.3390/antibiotics10070818/s1: Figure S1: FT-IR, ^1^H, ^13^C NMR and HRMS of compounds **3**–**8**.
