# Supplementary material for: In Vitro Antifungal Activity and Toxicity of Dihydrocarvone-Hybrid Derivatives against Monilinia fructicola
Source: Antibiotics (Basel). 2021 Jul 6;10(7):818. doi: 10.3390/antibiotics10070818 (PMC8300761; doi:10.3390/antibiotics10070818)

antibiotics

Communication

In Vitro Antifungal Activity and Toxicity of Dihydrocarvone-Hybrid Derivatives against *Monilinia fructicola*

Katy Díaz [1], Enrique Werner [2], Ximena Besoain [3], Susana Flores [4], Viviana Donoso [4], Bastian Said [5], Nelson Caro [6], Ernesto Vega [7], Iván Montenegro [8] and Alejandro Madrid [4,*]

[1] Departamento de Química, Universidad Técnica Federico Santa María, Av. España N° 1680, 2340000 Valparaíso, Chile; katy.diaz@usm.cl

[2] Departamento de Ciencias Básicas, Campus Fernando May, Universidad del Bío-Bío. Avda. Andrés Bello 720, casilla 447, 3780000 Chillán, Chile; ewerner@ubiobio.cl

[3] Escuela de Agronomía, Pontificia Universidad Católica de Valparaíso, San Francisco s/n La Palma, 2260000 Quillota, Chile; ximena.besoain@pucv.cl

[4] Laboratorio de Productos Naturales y Síntesis Orgánica (LPNSO), Departamento de Química, Facultad de Ciencias Naturales y Exactas, Universidad de Playa Ancha, Avda. Leopoldo Carvallo 270, Playa Ancha, 2340000 Valparaíso, Chile; susana.flores@upla.cl (S.F.); cadonoso@utalca.cl (V.D.)

[5] Departamento de Química, Universidad Técnica Federico Santa María, Av. Santa María 6400, 7630000 Santiago, Chile; bastian.said@usm.cl

[6] Centro de Investigación Australbiotech, Universidad Santo Tomás, Avda. Ejército 146, 8320000 Santiago, Chile; ncaro@australbiotech.cl

[7] Departamento Laboratorios y Estaciones Cuarentenarias, Servicio Agrícola y Ganadero, Ruta 68 # (Km. 12), 19100 Pudahuel, Santiago, Chile; ernesto.vega@sag.gob.cl

[8] Escuela de Obstetricia y Puericultura, Facultad de medicina, Universidad de Valparaíso, Angamos 655, 2520000 Reñaca, Viña del Mar, Chile; ivan.montenegro@uv.cl

* Correspondence: alejandro.madrid@upla.cl; Tel.: +56-(032)-250-0526

Figure S1：FT-IR, ^{1}H, ^{13}C NMR and HRMS of compounds 3–8

FT-IR of compounds 3

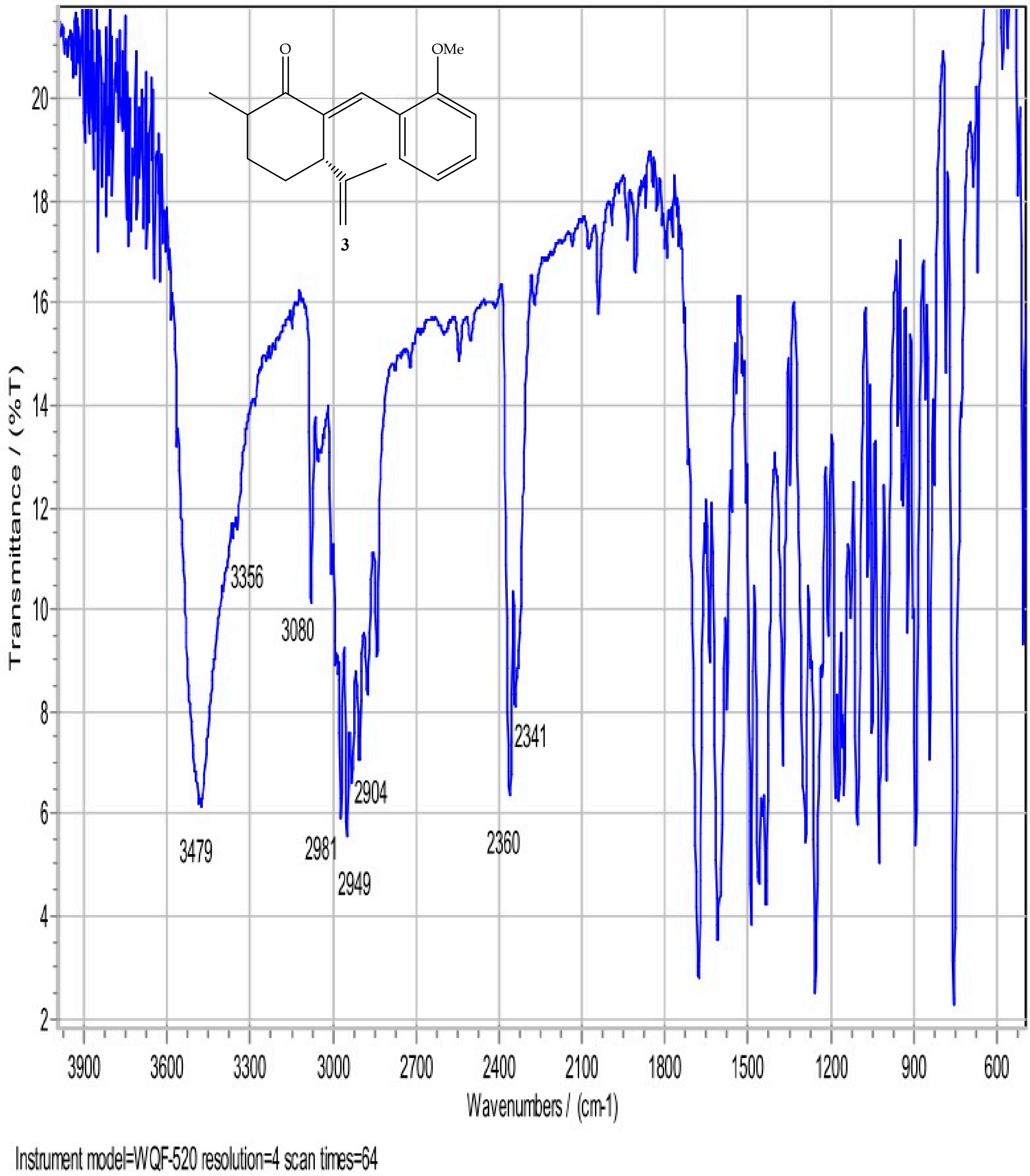

^{1}H NMR (400 MHz, $CDCl_3$) spectrum of compound 3

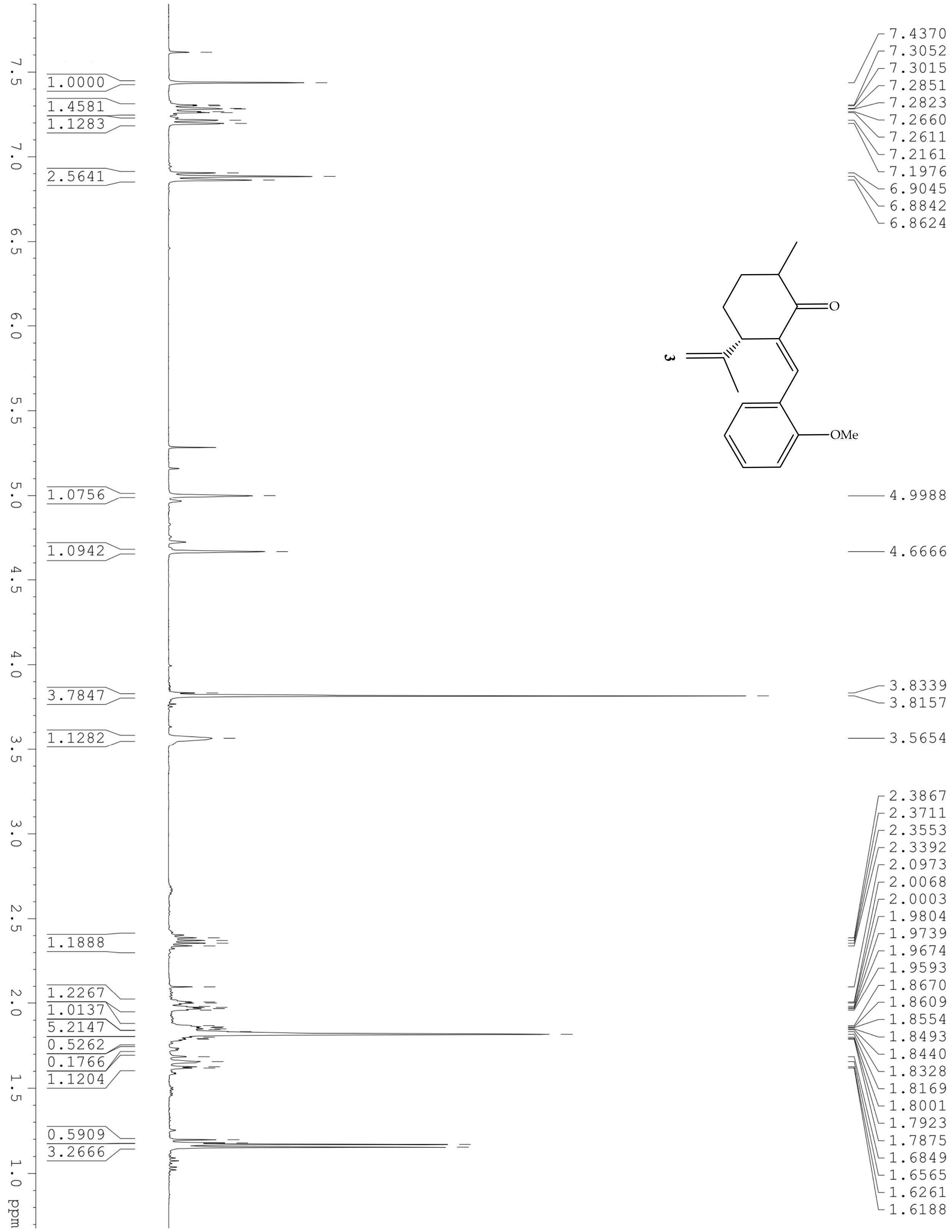

^{13}C NMR (100 MHz, $CDCl_3$) spectrum of compound 3

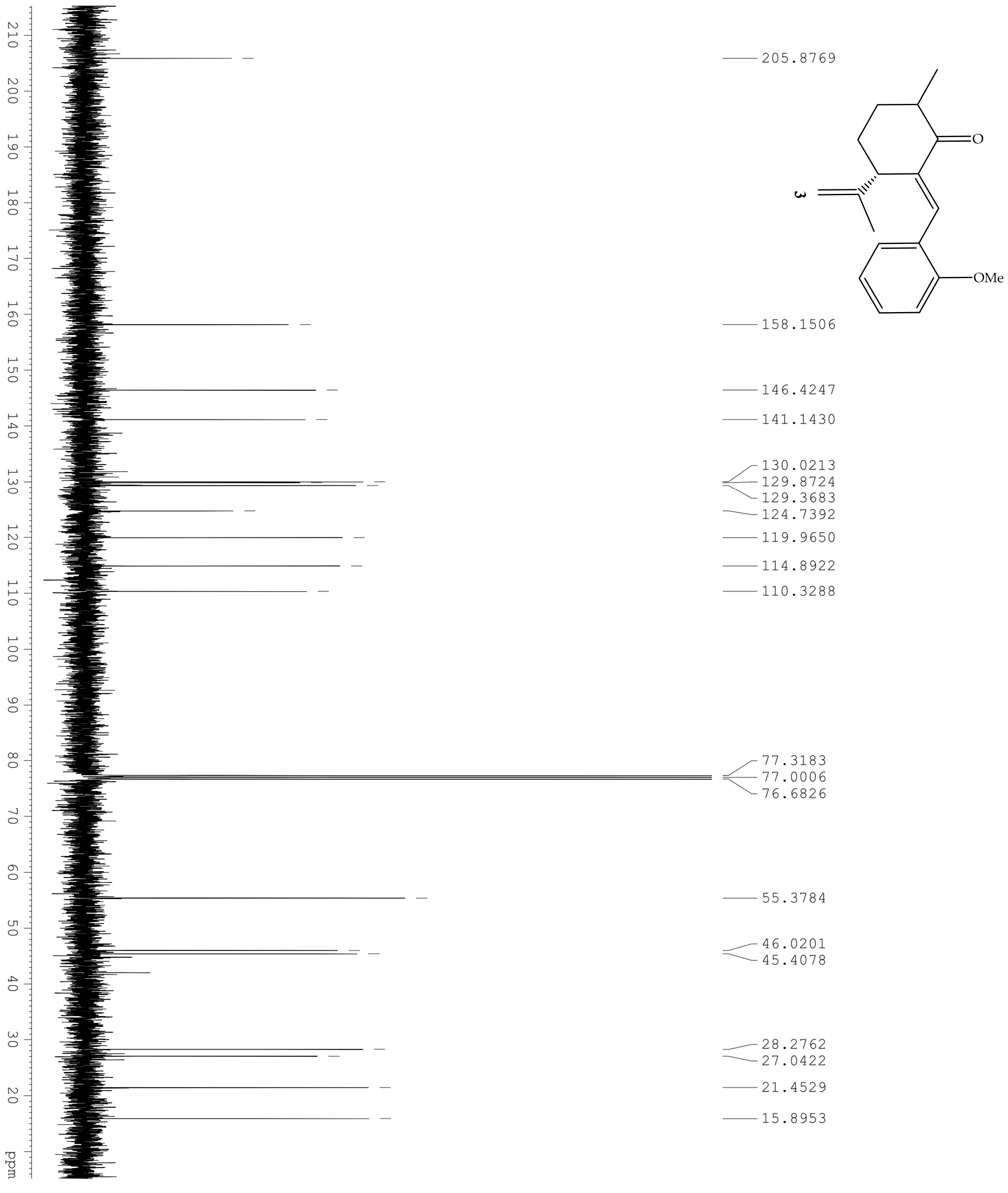

HRMS of compound 3

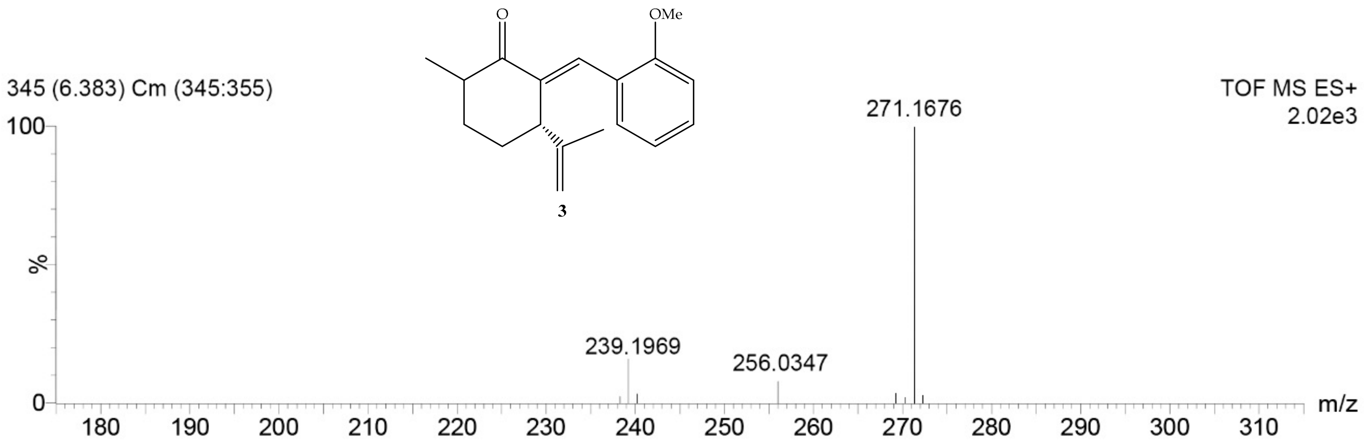

FT-IR of compounds 4

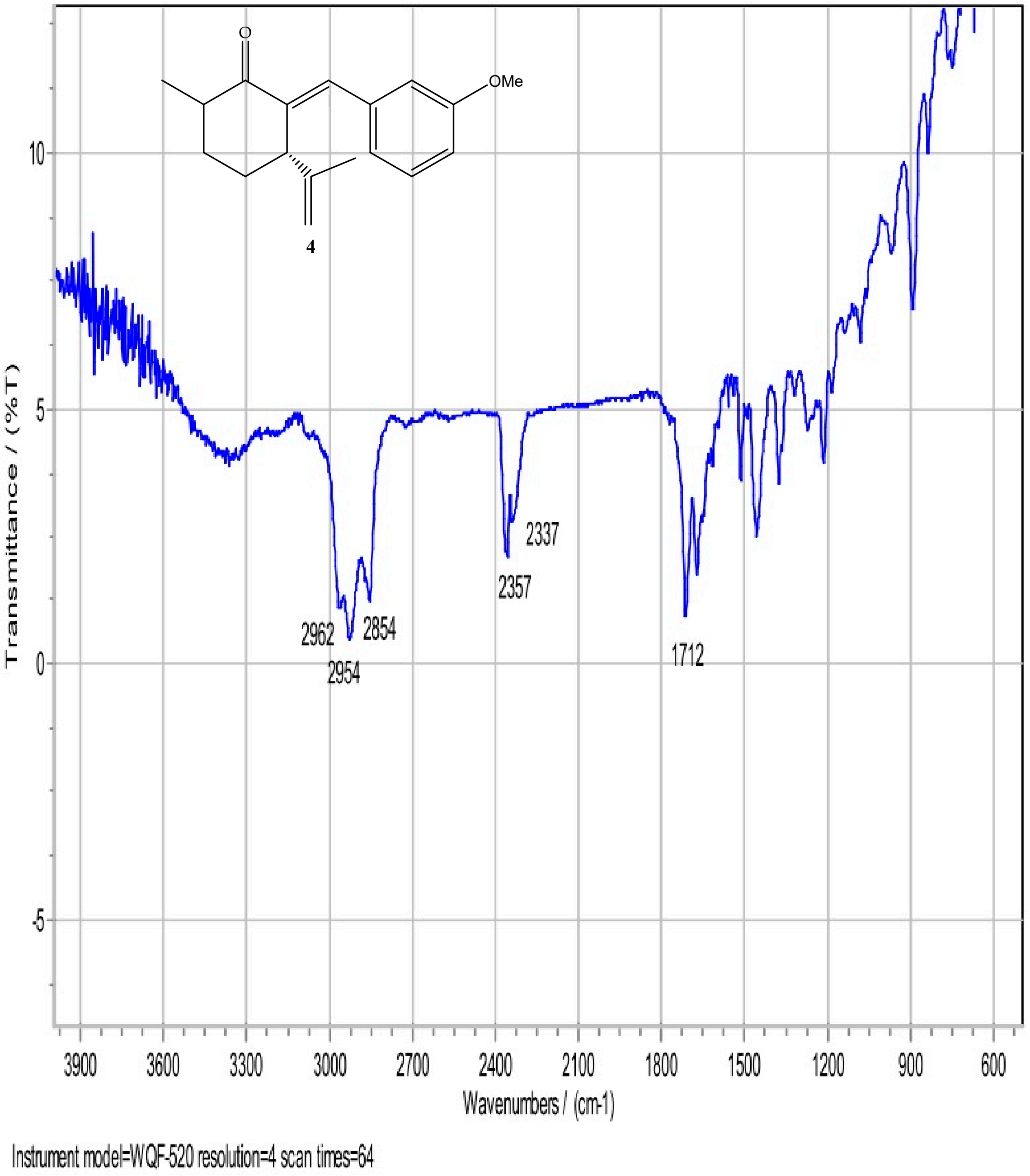

^{1}H NMR (400 MHz, CDCl₃) spectrum of compound 4

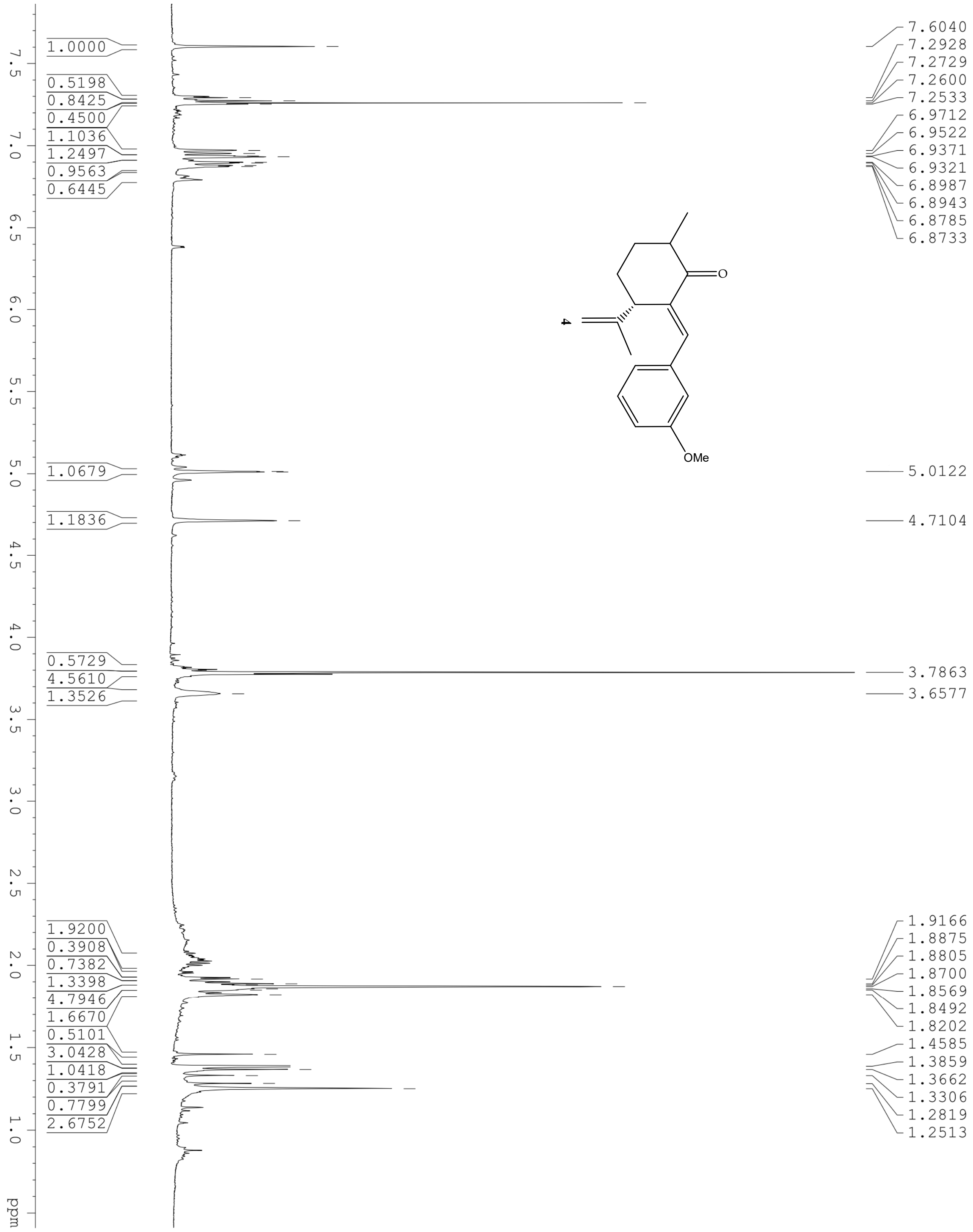

^{13}C NMR (100 MHz, CDCl3) spectrum of compound 4

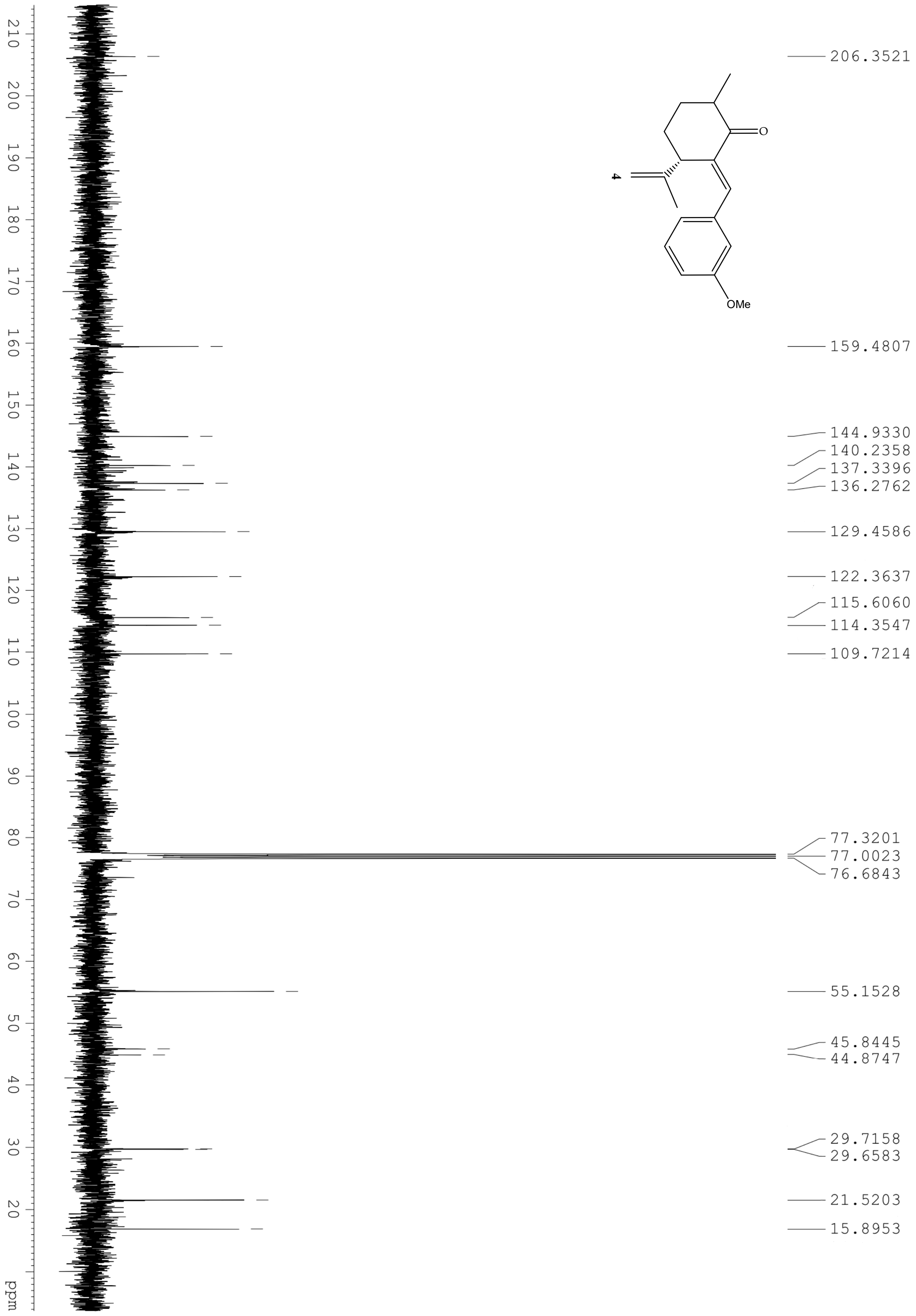

HRMS of compound 4

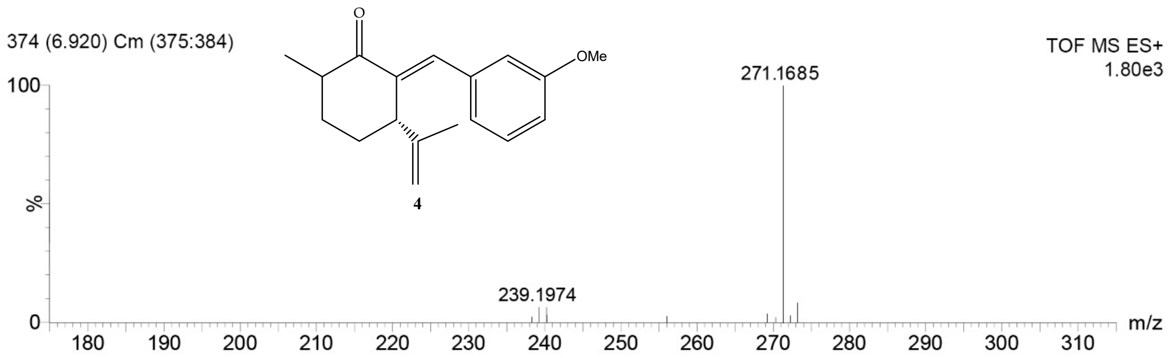

FT-IR of compounds 5

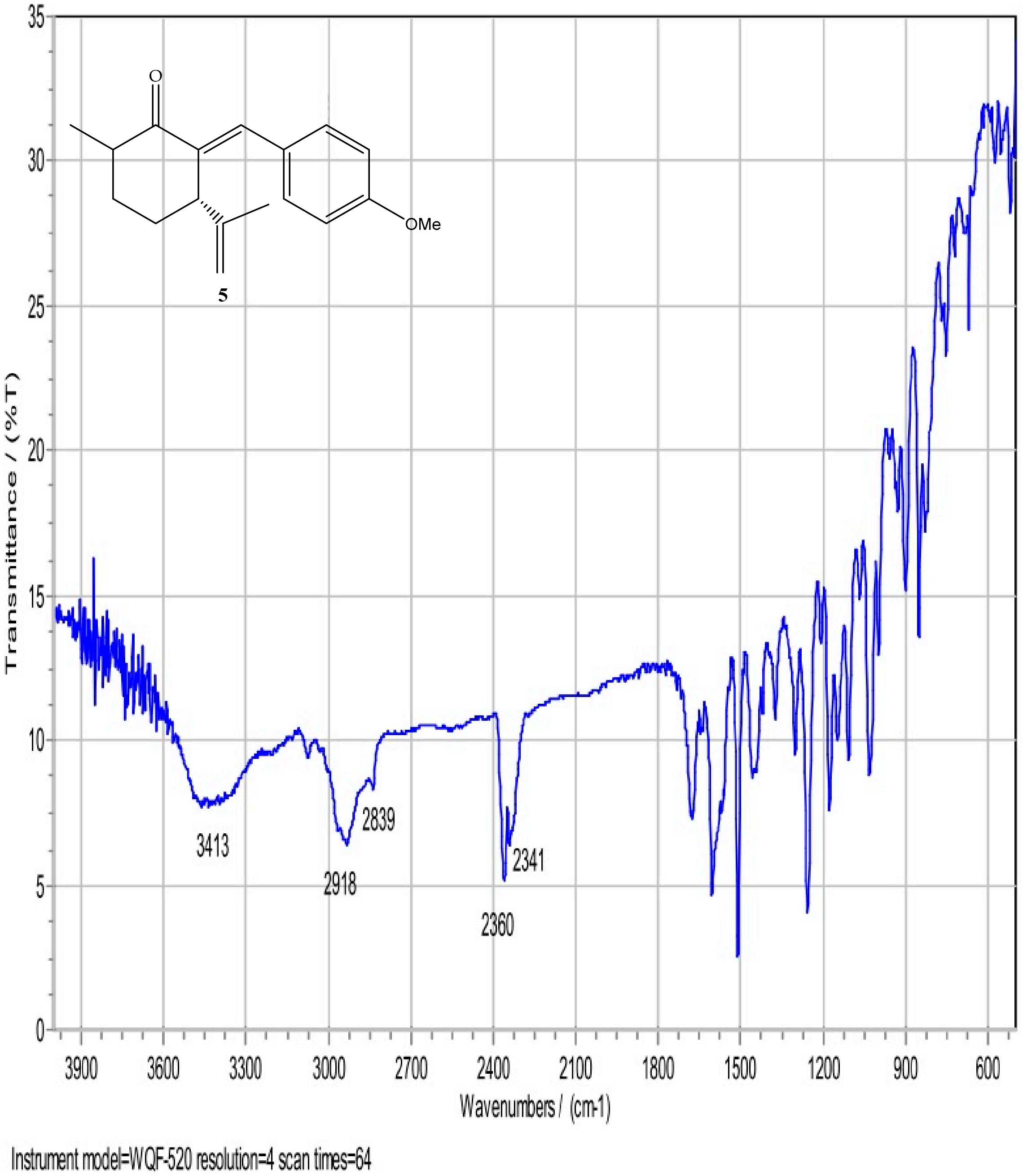

^{1}H NMR (400 MHz, $CDCl_3$) spectrum of compound 5

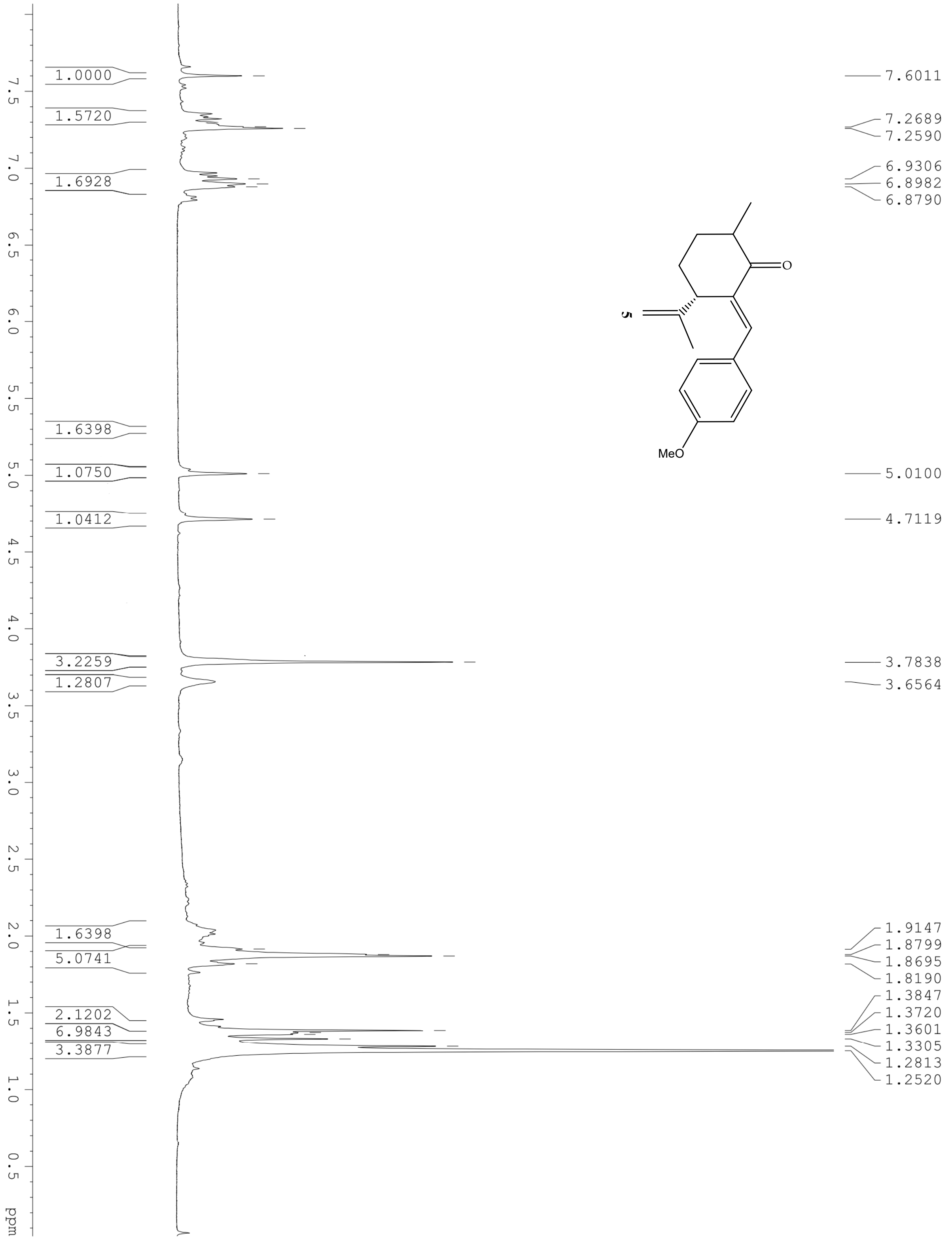

^{13}C NMR (100 MHz, CDCl$_3$) spectrum of compound 5

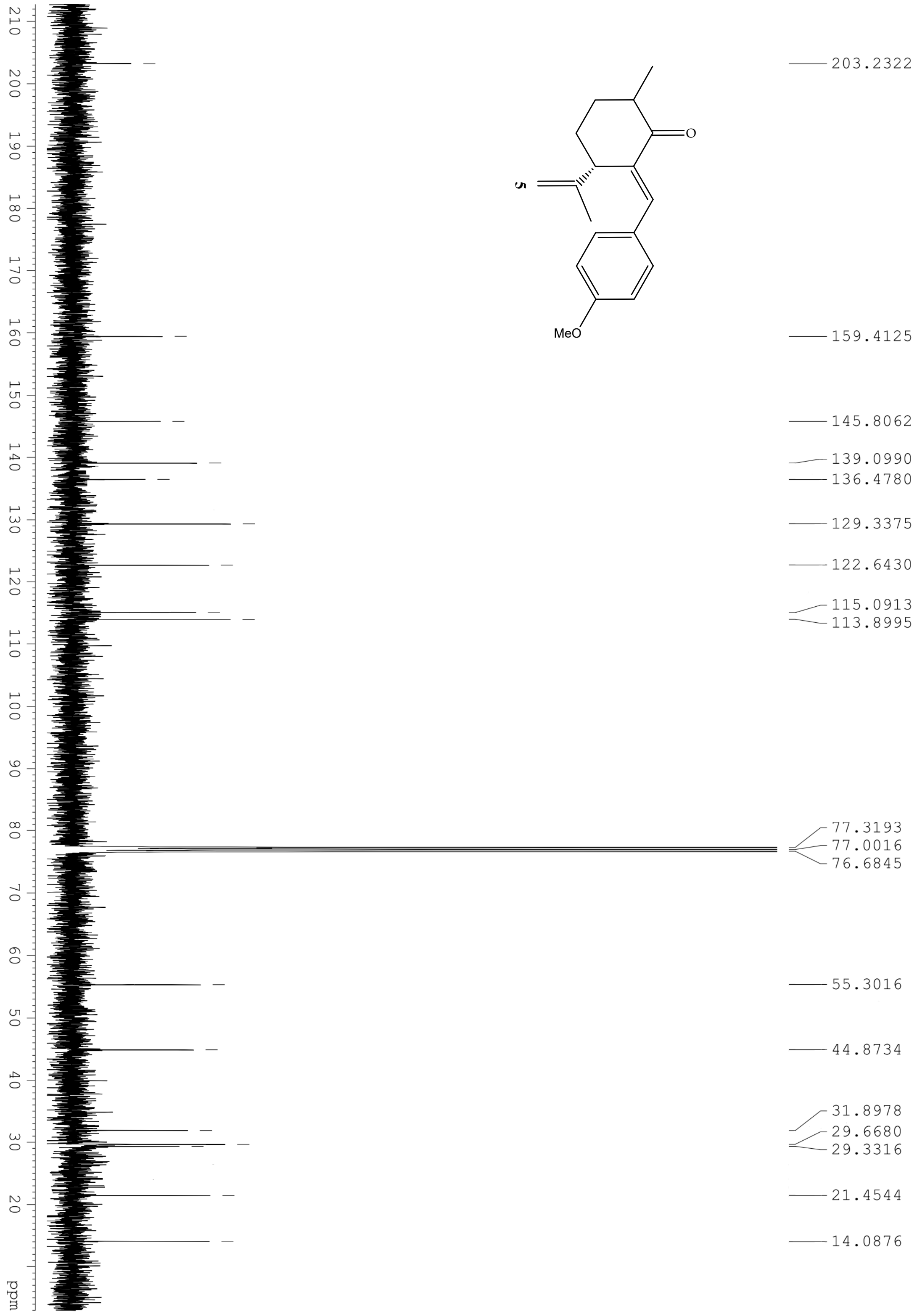

HRMS of compound 5

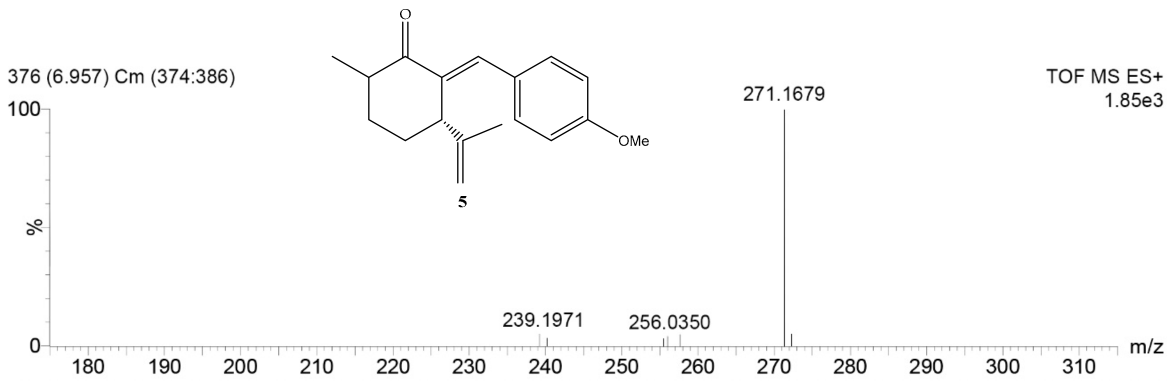

FT-IR of compounds 6

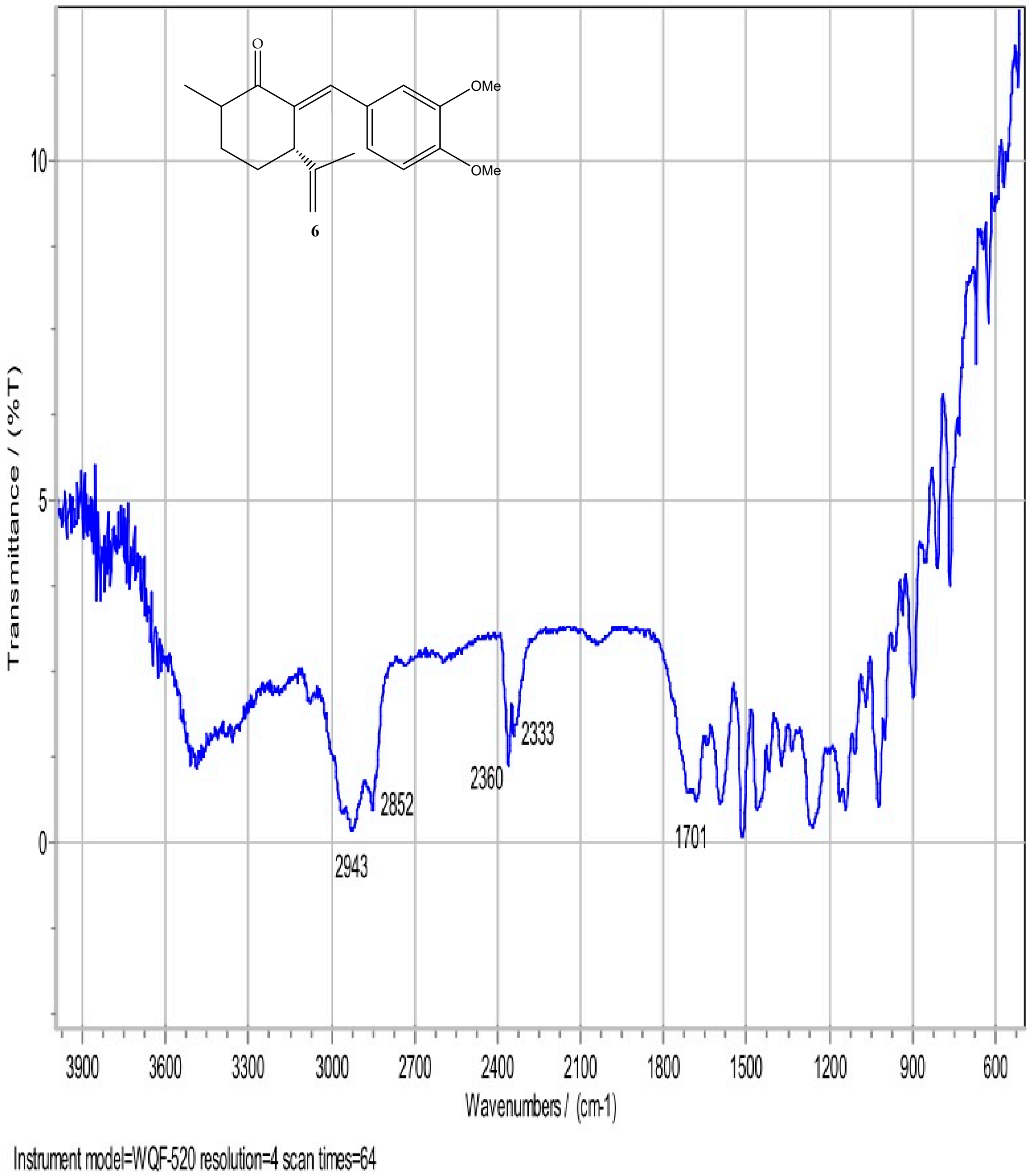

^{1}H NMR (400 MHz, $CDCl_3$) spectrum of compound 6

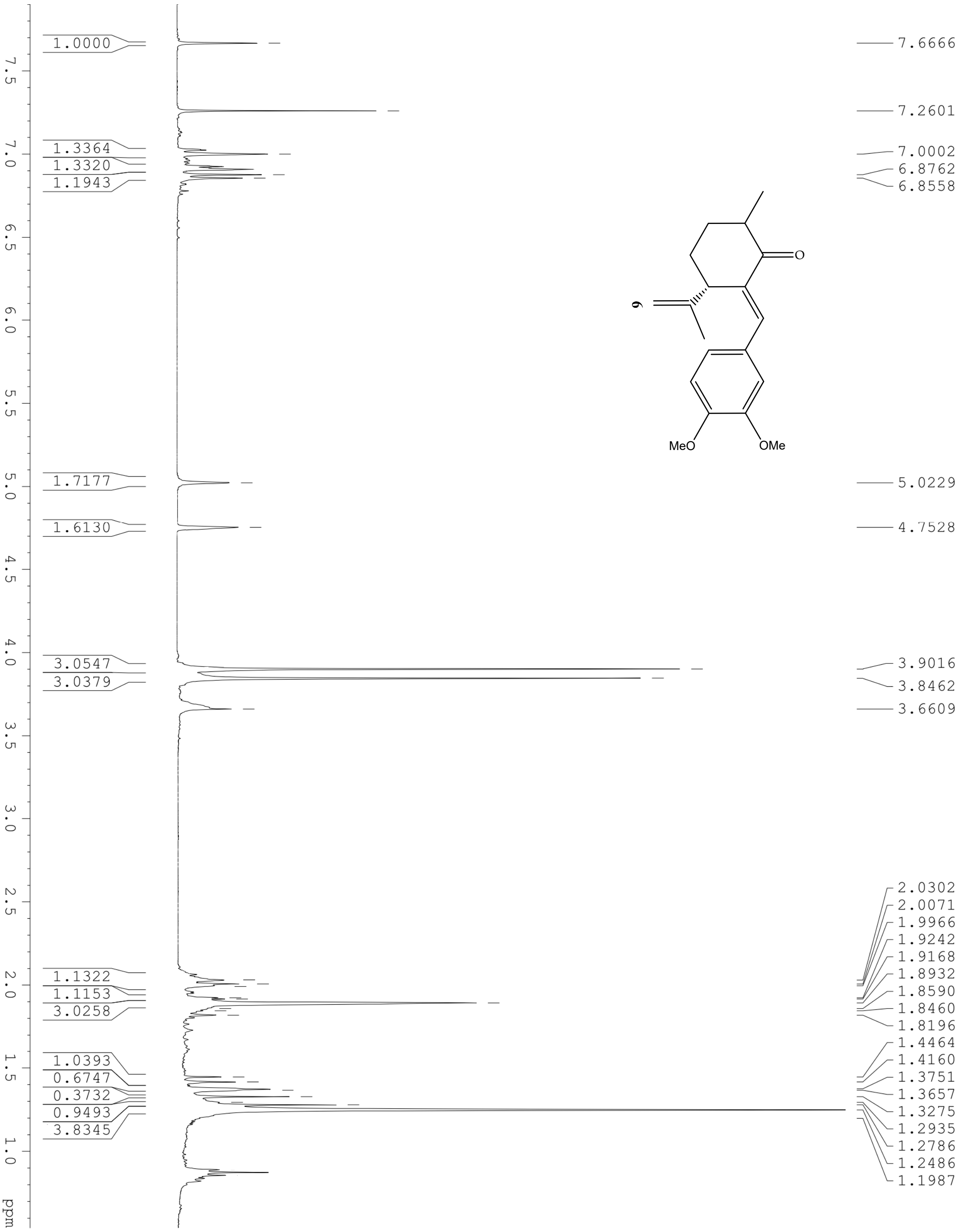

^{13}C NMR (100 MHz, $CDCl_3$) spectrum of compound 6

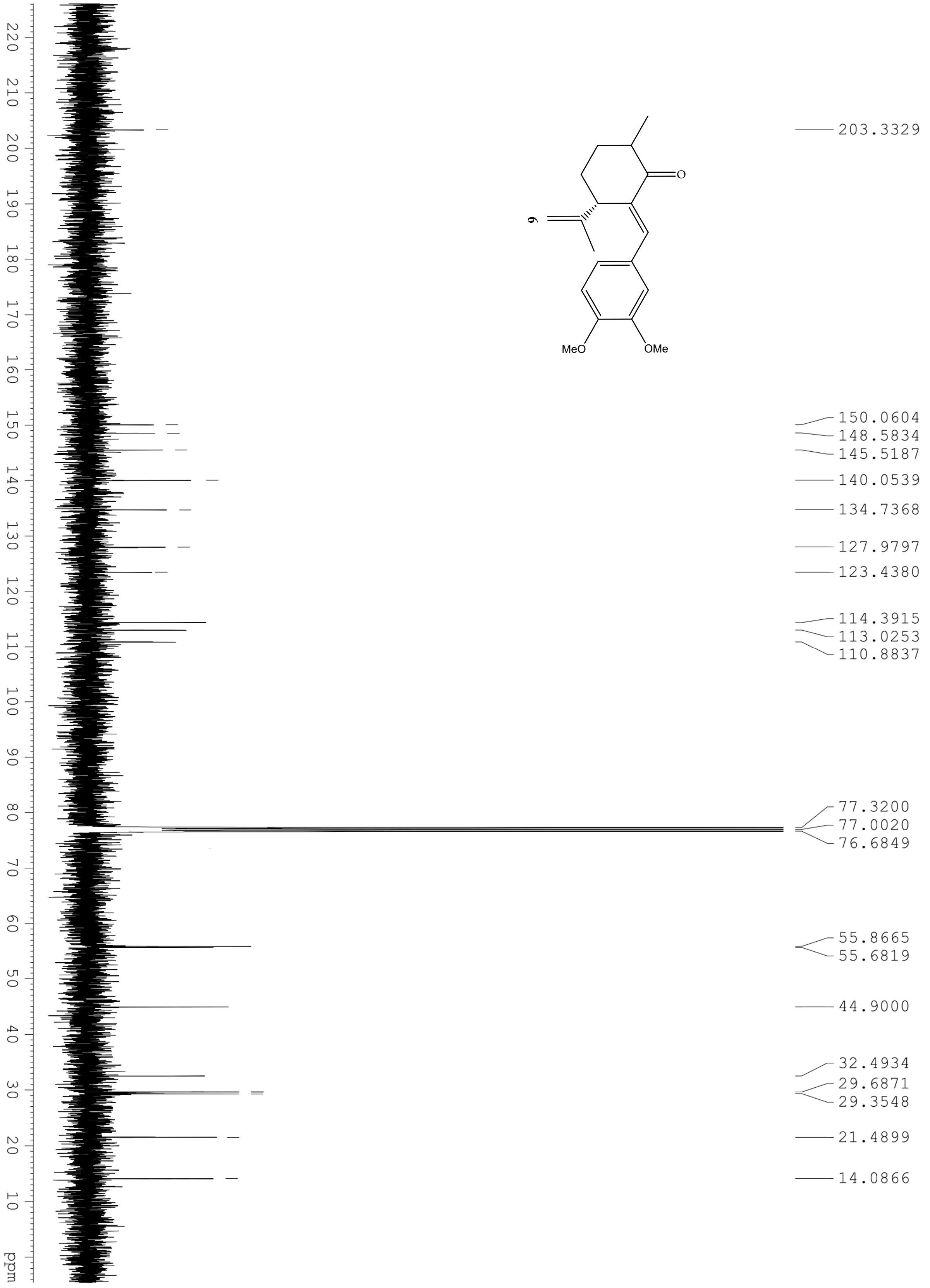

HRMS of compound 6

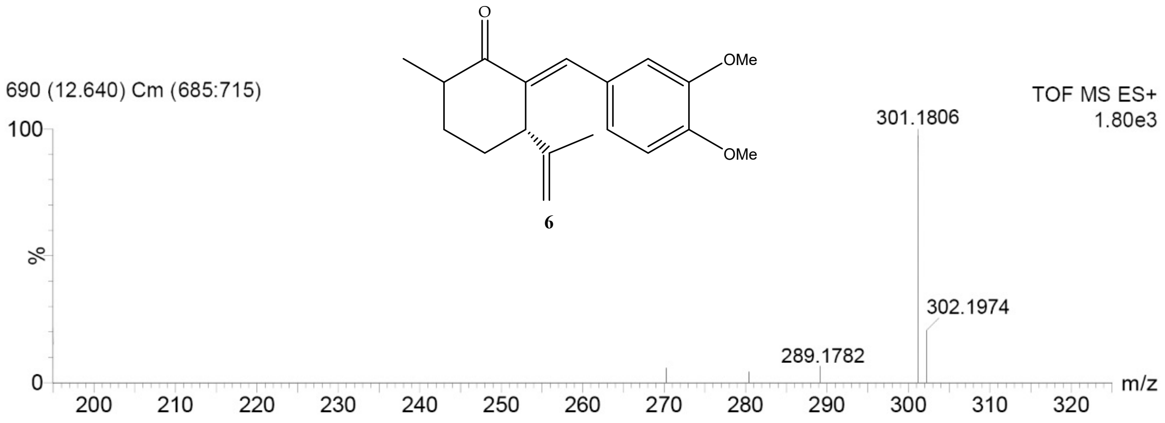

FT-IR of compounds 7

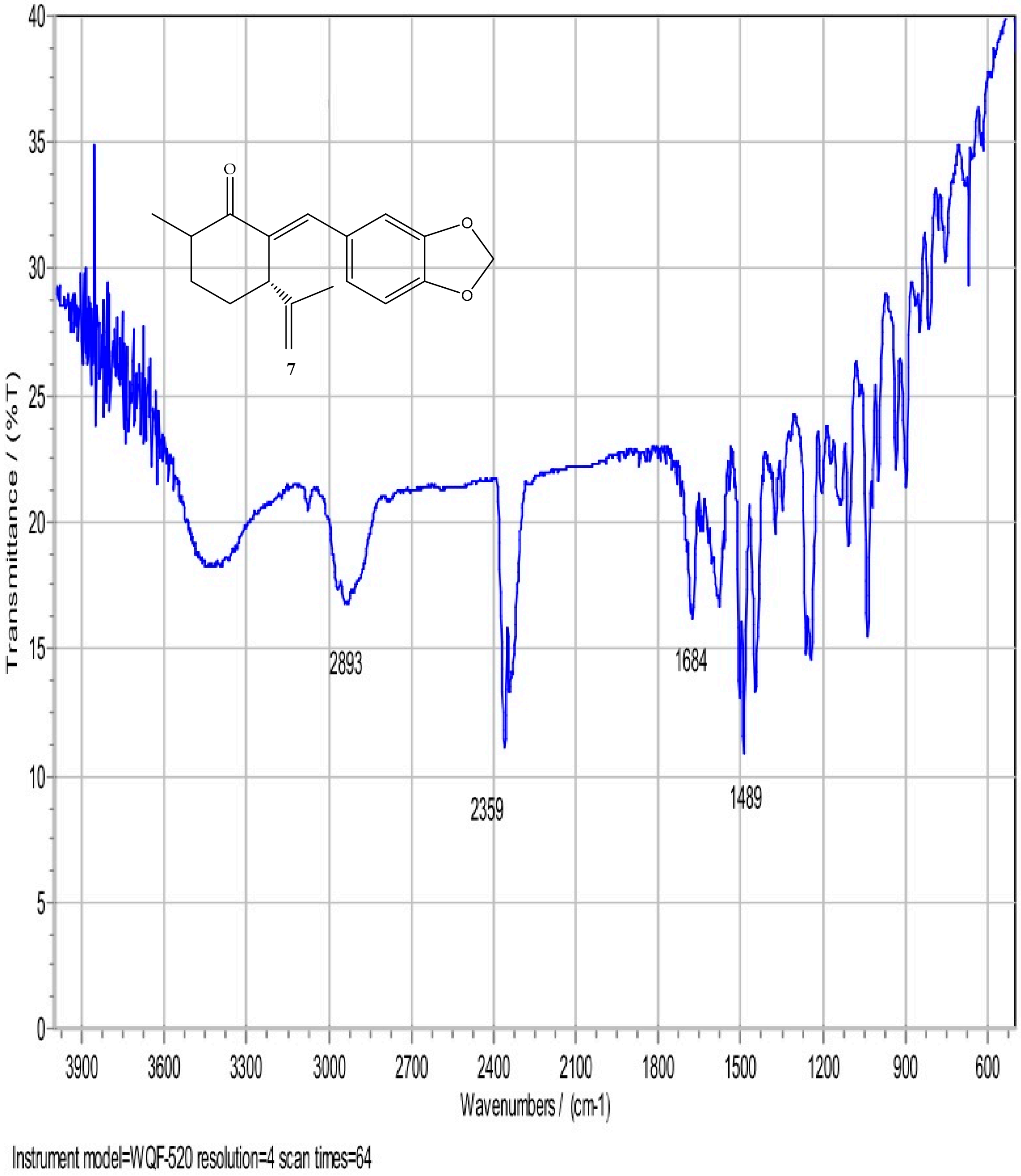

^{1}H NMR (400 MHz, $CDCl_3$) spectrum of compound 7

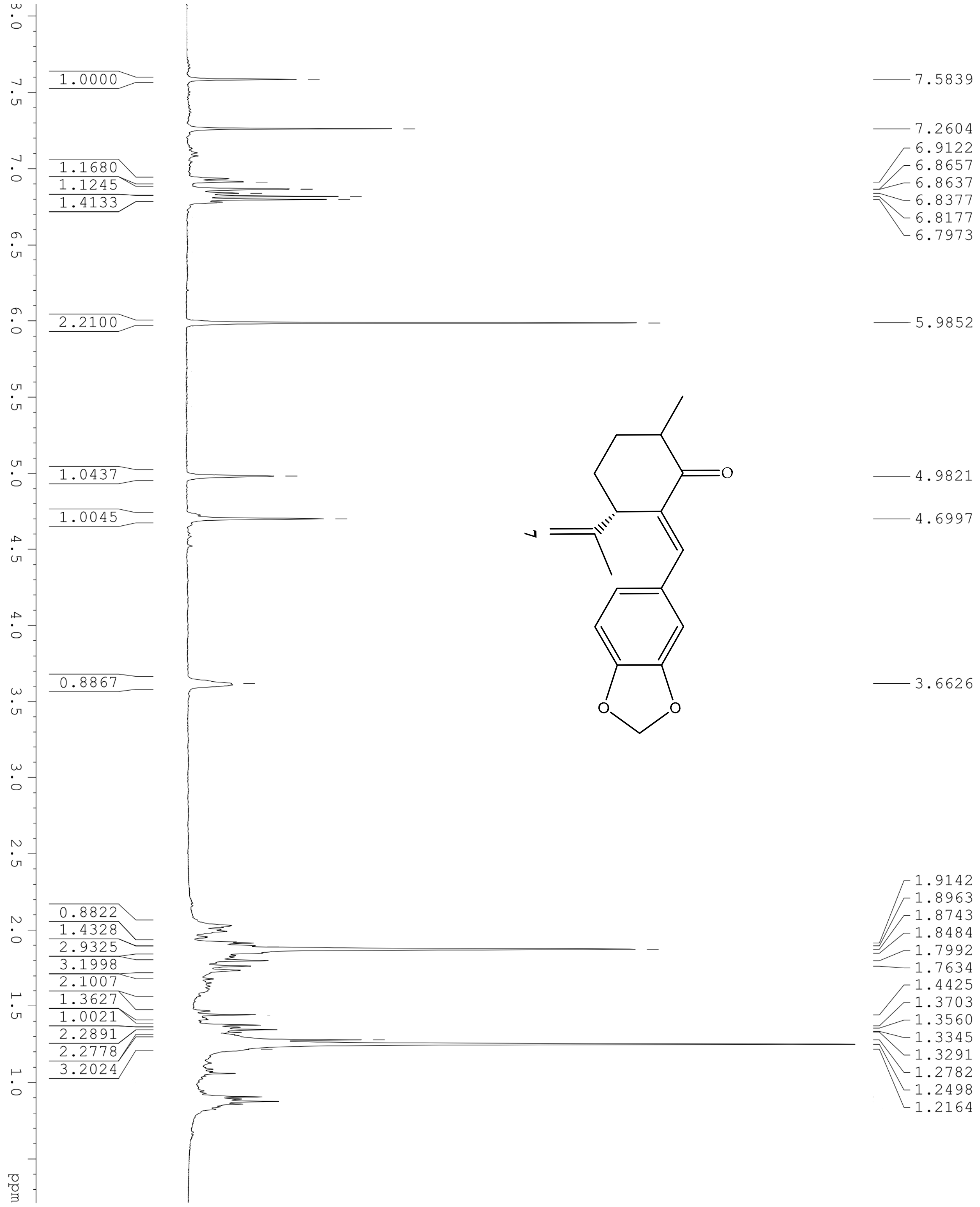

^{13}C NMR (100 MHz, $CDCl_3$) spectrum of compound 7

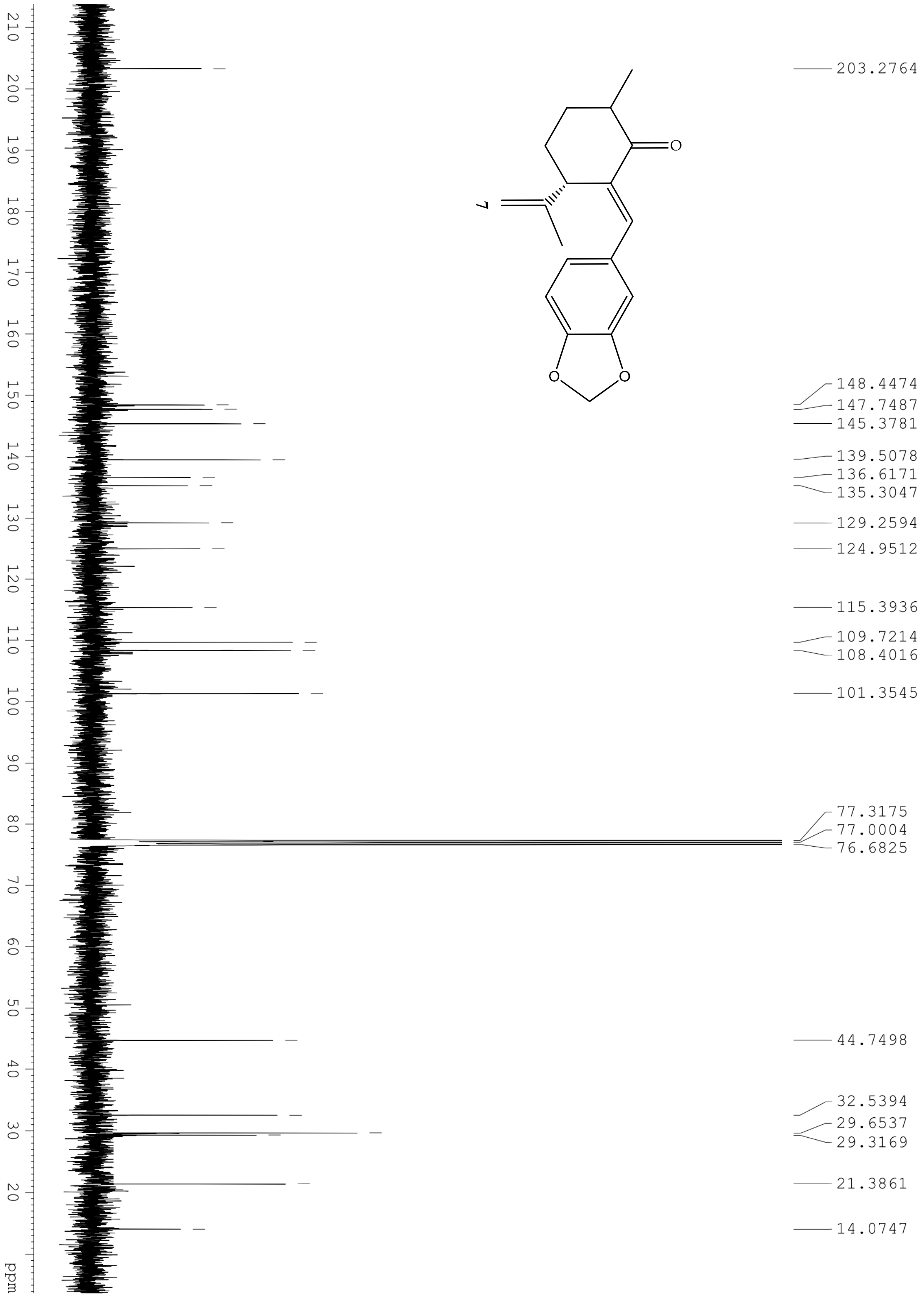

HRMS of compound 7

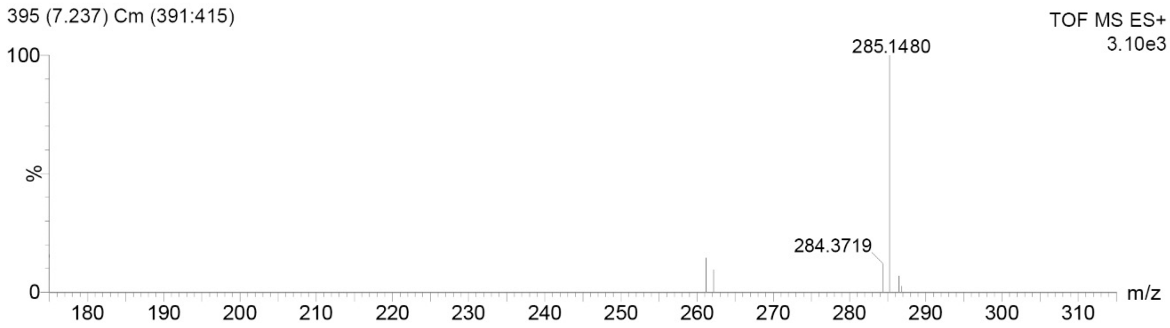

FT-IR of compounds 8

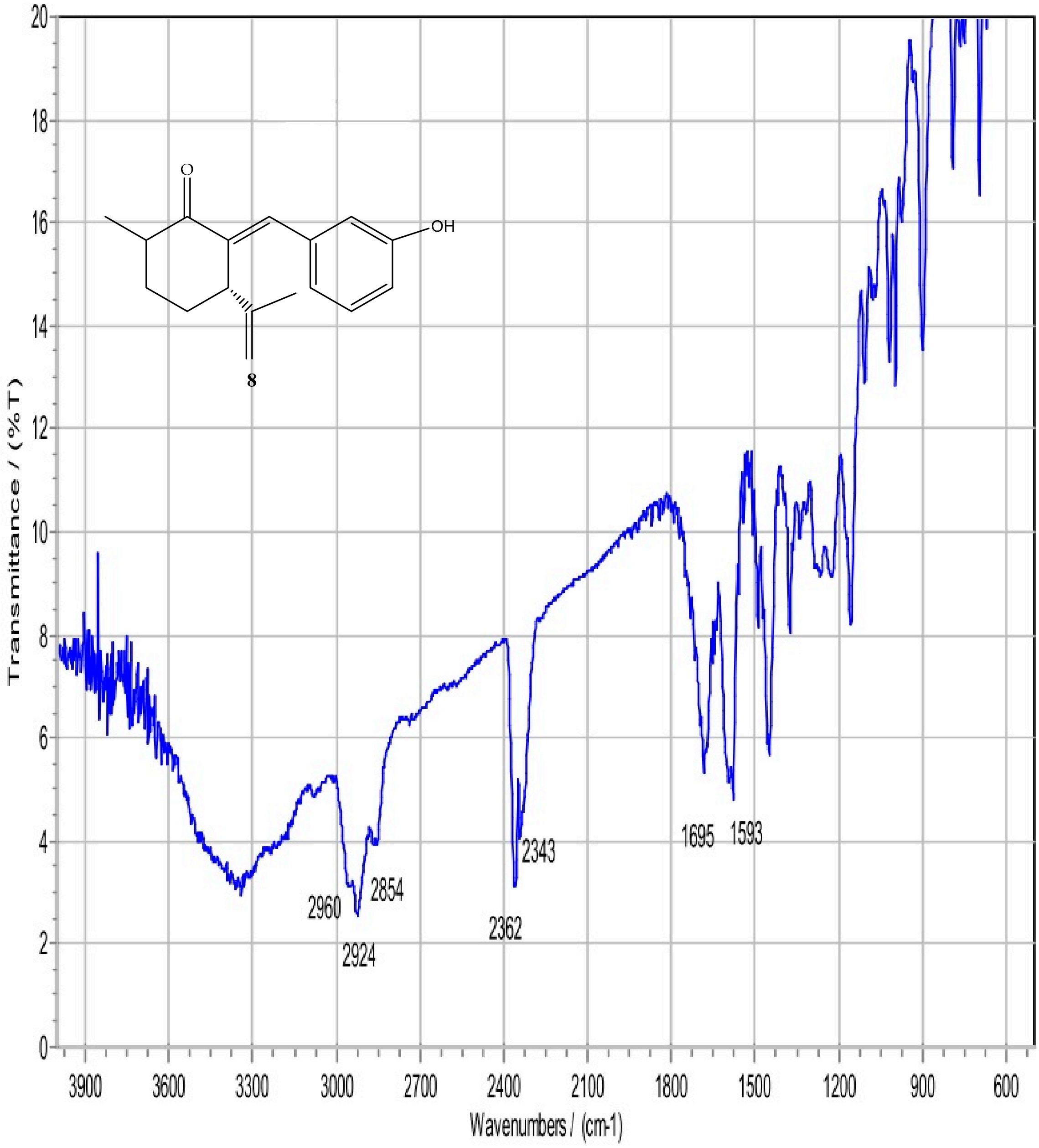

Instrument model=WQF-520 resolution=4 scan times=64

^{1}H NMR (400 MHz, $CDCl_3$) spectrum of compound 8

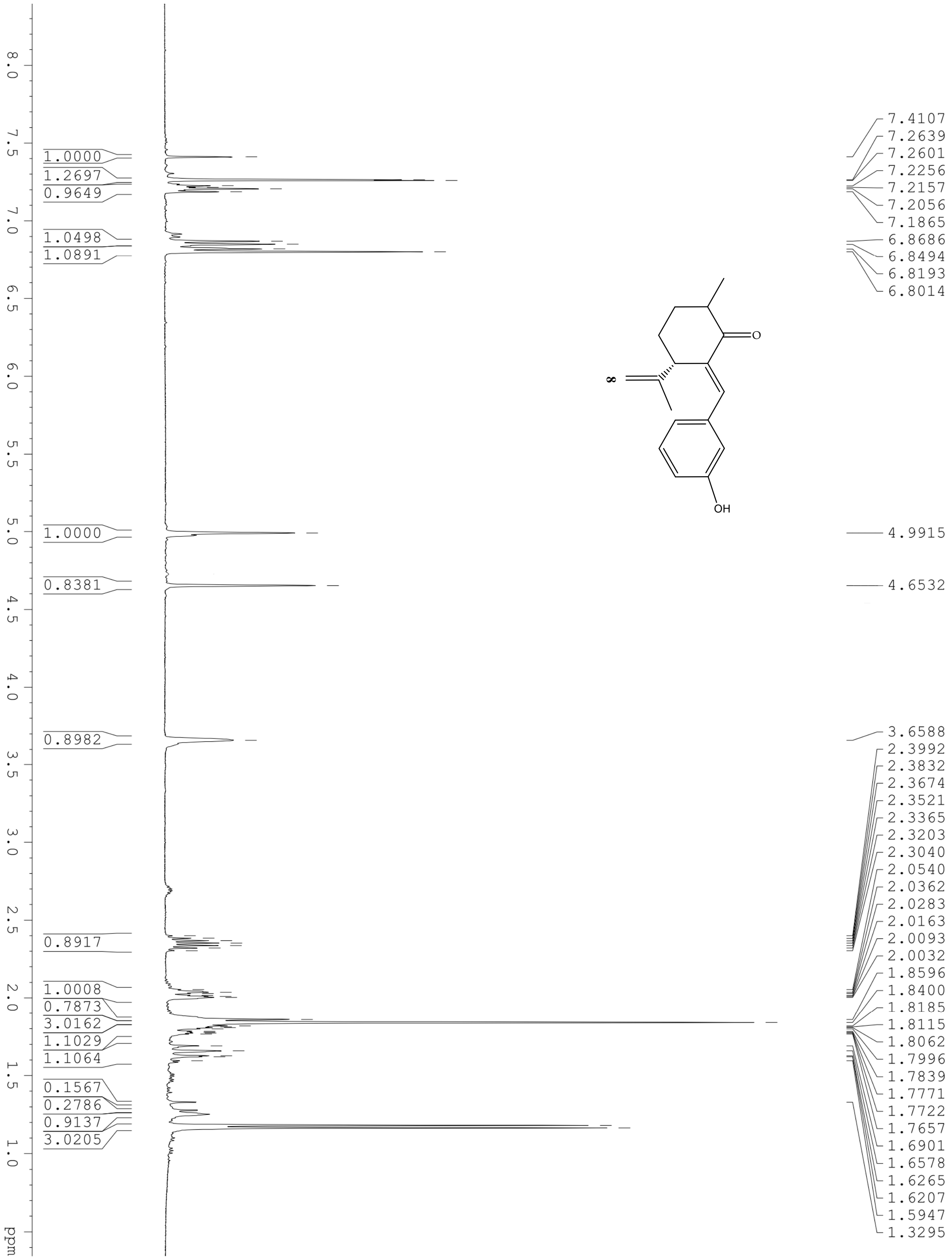

^{13}C NMR (100 MHz, $CDCl_3$) spectrum of compound 8

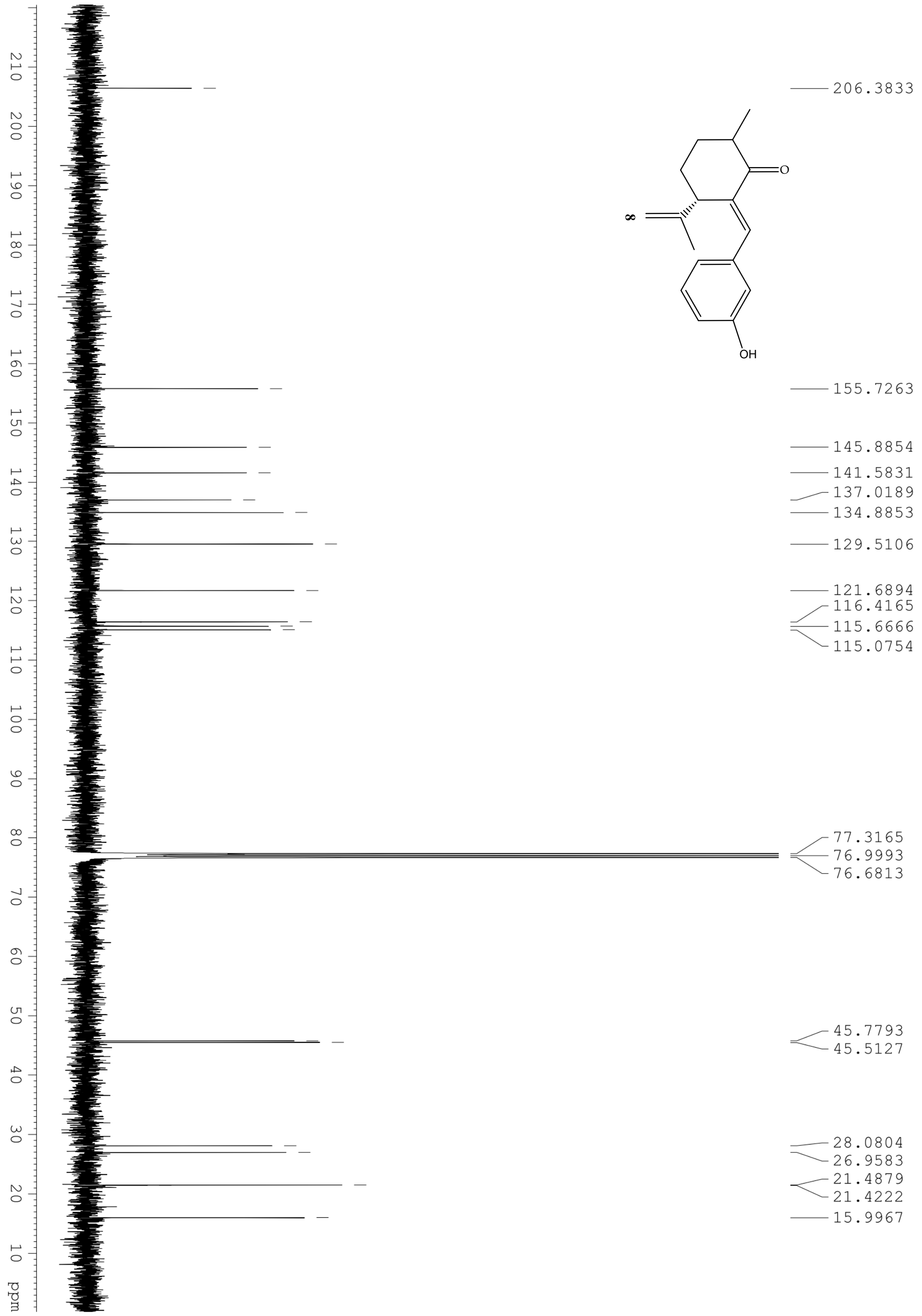

HRMS of compound 8

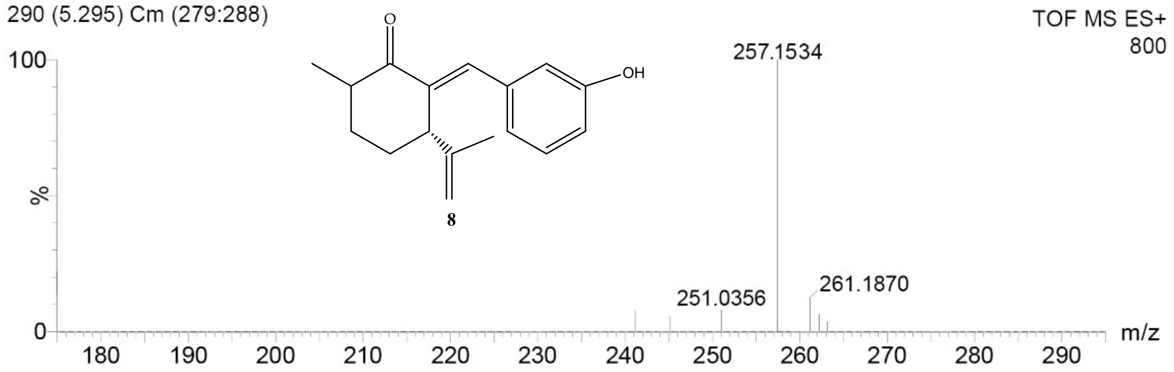

Supplement: Supplementary file 1 [file antibiotics-10-00818-s001.zip › antibiotics-1268959--supplementary-final.pdf]
